# YY1 Lactylation Elicits CARD9 Deficiency in Dendritic Cells Promoting Pancreatic Cancer Immune Escape

**DOI:** 10.7150/ijbs.131102

**Published:** 2026-05-01

**Authors:** Lingyan Ding, Cheng Tian, Yiting Feng, Ruochen Xu, Senlin Li, Mengzhu Zheng, Xiaoyan Wang, Qianqian Xu, Ming Xiang

**Affiliations:** 1 Department of Pharmacology, School of Pharmacy, Tongji Medical College, Huazhong University of Science and Technology, Wuhan 430030, China.; 2 Department of Pharmacy, Tongji Hospital, Tongji Medical College, Huazhong University of Science and Technology, Wuhan 430022, China.; 3 Department of Pharmacy, Union Hospital, Tongji Medical College, Huazhong University of Science and Technology, Wuhan 430022, China.; 4 Institute of Pharmaceutical Innovation, Hubei Province Key Laboratory of Occupational Hazard Identification and Control, School of Medicine, Wuhan University of Science and Technology, Wuhan 430065, China.; 5 Department of Pathology, Tongji Hospital, Tongji Medical College, Huazhong University of Science and Technology, Wuhan 430030, China.; 6 Department of Pharmacy, Maternal and Child Health Hospital of Hubei Province, Tongji Medical College, Huazhong University of Science and Technology, Wuhan 430070, China.

**Keywords:** CARD9, dendritic cell, pancreatic cancer, YY1, lactylation

## Abstract

Pancreatic cancer (PC) cells suppress dendritic cell (DC) maturation and function through multiple pathways, further impairing antitumor activity of CD8^+^ T cells. Our previous study revealed that caspase-recruitment domain-containing protein 9 (CARD9) deficiency led to DC dysfunction and exacerbated PC progression, yet the precise mechanism underlying CARD9 downregulation in DCs remains elusive. In this study, we observed that CARD9 expression was progressively downregulated in PC tumors and associated with advanced clinicopathological stages and DC dysfunction. Functionally, PC cells reduced CARD9 expression, impaired DC maturation and weakened CD8^+^ T cell activation, with these suppressive effects attenuated in CARD9-deficient DCs and reversed when CARD9 was restored. Then, we identified YY1 as a critical upstream transcription factor that bound to the promoter of CARD9 and repressed it, accompanied with DC dysfunction and tumor growth. Mechanistically, tumor-derived lactate induced YY1 lactylation at lysine 183, facilitating YY1 nuclear entry and strengthening its combination with CARD9 promoter. Furthermore, we identified p300 as the “writer” catalyzing YY1-K183 lactylation, and HDAC2 as its enzymatic “eraser”. Lactylation enhanced YY1 stability by limiting ubiquitination, sustaining YY1 activation and reinforcing CARD9 suppression. Overall, our findings define a lactate-p300/HDAC2-YY1 lactylation-CARD9 regulatory axis that restricts DC function and promotes immune escape in PC.

## Introduction

Pancreatic cancer (PC) remains one of the most lethal malignancies, with a five-year survival rate of only 13%, among which, pancreatic ductal adenocarcinoma (PDAC) comprises more than 95% of all cases^1^. The dismal prognosis of PC is largely attributed to its inherently aggressive biology, the absence of effective therapeutic strategies, and its asymptomatic onset, which frequently leads to diagnosis at an advanced stage^2^. As the principal antigen-presenting cells in the tumor, DCs orchestrate antitumor immunity by modulating the magnitude and persistence of infiltrating cytotoxic T lymphocyte responses^3^. However, their scarcity within the pancreatic tumor microenvironment (TME) limits T-cell priming and shapes an immune “cold” phenotype^4^.

Caspase-recruitment domain-containing protein 9 (CARD9) is a pivotal adaptor molecule that bridges innate and adaptive immune responses. It is predominantly expressed in myeloid cells, particularly DCs and macrophages^5^. Increasing investigations indicate that CARD9 engages in diverse physiological and pathological processes, such as inflammatory bowel disease, lung cancer, and cardiovascular disease^6^-^9^. Our prior work also revealed that CARD9 deficiency inhibits the CBM-p65-SLC6A8 axis, leading to defective creatine transfer to DCs, impaired DC function and weakened CD8^+^ T cell-mediated antitumor immunity, thereby aggravating PC progression^10^. Nevertheless, the mechanisms underlying the CARD9 deficiency in DCs warrant further elucidation. Delineating these mechanisms may uncover new tumor immunity pathways and offer promising strategies to enhance immunotherapy efficacy in PC.

Yin Yang 1 (YY1) is a zinc finger transcription factor that functions as both a transcriptional activator and repressor^11^. Accumulating evidence has reinforced that YY1 is aberrantly upregulated in several malignancies, including ovarian, breast, cervical cancers, and osteosarcoma^12^-^15^. However, its role in PC remains poorly understood. Metabolic reprogramming is a hallmark of cancer^16^. Increased glycolysis and lactate production are commonly observed in solid tumors, leading to the formation of an acidic tumor microenvironment^17^. Recently, lysine lactylation has been identified as a novel post-translational modification (PTM) derived from lactate, a byproduct of cellular metabolism. This modification transforms our perception to a crucial regulator of tumor angiogenesis, metastasis and antitumor immunity^18^-^20^. Notably, non-histone lactylation has been shown to profoundly influence tumor metabolism, proliferation and invasion, offering new insights into immune evasion mechanisms and potential therapeutic targets in cancer immunotherapy^21^-^23^. Despite its significance, whether intracellular lactate modulates DC function remains unclear.

Here, we disclosed a specific mechanism by which PC cells release lactate to induce YY1 lactylation at the K183 site through the coordinated actions of p300 and HDAC2. This modification enhances YY1 transcriptional activity and promotes its nuclear localization. Activated YY1 directly binds to the CARD9 promoter, suppressing CARD9 expression and impairing DC function, thereby facilitating immune escape in PC. Altogether, these findings delineate a previously unrecognized molecular pathway underlying CARD9 attenuation in DCs and highlight a promising framework for developing strategies to overcome immune evasion in PC.

## Materials and Methods

### Cell culture

Murine pancreatic adenocarcinoma cells Panc02 were purchased from Shanghai Aoru Biotechnology Co. Ltd and cultured in high-glucose DMEM (89% DMEM + 10% fetal bovine serum + 1% penicillin-streptomycin double antibiotic), and placed in a constant temperature incubator (5% CO_2_) at 37°C with humidified sterile water.

### Mice

6-8 weeks SPF grade wild-type (WT) C57BL/6J male mice (20 ± 2 g) were purchased from Beijing Viton Lihua Laboratory Animal Technology Co (Beijing, China). CARD9^-/-^ mice and CD11c-diphtheria toxin receptor (DTR) mice on C57BL/6J background were obtained from Guangzhou Saiye Biotechnology Co (Guangzhou, China). All mice were housed in the SPF Grade Laboratory Animal Center of Tongji Medical College, Huazhong University of Science and Technology. Mice were acclimatized for 7 days prior to the experiment in an environment with a temperature of 25 ± 2 ℃, humidity of 50-60%, a 12 h light/dark cycle, and free access to food and water. All experimental procedures were approved by the Institutional Animal Care and Use Committee of Tongji Medical College (IACUC Number: 4253).

### Clinical tissue samples

Twenty-seven pairs of paraffin-embedded PC and para-cancer tissue samples (9 female and 18 male patients, mean age: 58.2 ± 14.9 years) were from the Department of Pathology, Tongji Hospital of Huazhong University of Science and Technology. All patients signed consent before operation. This study was approved by Ethics Committee of Tongji Hospital (Number TJ-IRB202407046). Researchers were blinded to the group allocation both during the experiment and/or when assessing the outcome.

### Extraction, induction and processing of mouse bone marrow-derived DCs

Mouse bone marrow-derived DCs (BMDCs), obtained from WT or CARD9^-/-^ mice bone marrow cultured in RPMI-1640 complete medium (~2 × 10^6^ cells/mL), and granulocyte macrophage colony-stimulating factor (20 ng/mL, PeproTech), and interleukin (IL)-4 (10 ng/mL, PeproTech) were added during culture. After half-exchange of fluid on day 3 and 5, it was replaced with normal RPMI-1640 complete medium on day 7, while LPS (250 ng/mL, Sigma) was added to treat the DCs with pancreatic tumor antigens for 24 h to induce maturation of loaded tumor antigens.

### Mixed lymphocyte reaction (MLR)

Isolated CD3^+^ T cells were co-cultured with different groups of DCs in a 10:1 ratio for 72 h. Monensin (5 µM, MedChemExpress) was added 4 h before the end of the co-culture to inhibit IFN-γ release. Proportion of CD8^+^ T cells and degree of activation were detected by APC-IFN-γ and PE-CD8 flow antibody incubation.

The isolated CD3^+^ T cells were incubated with CFSE for 15 min, and then co-cultured with different groups of DCs at a ratio of 10:1 for 72 h. The proliferation status of T cells was detected on the machine.

### Flow cytometry

Flow cytometry was performed using Sony ID7000 (Sony, Tokyo, Japan) or C6 flow cytometer (BD Accuri), and the data were processed via FlowJo 10. Samples were stained with fluorescent dye-labeled antibodies for 60 min at 4°C under dark conditions. Proteins within the cell membrane were membrane-broken using a cell fixation/permeabilization membrane-breaking kit prior to antibody incubation.

Fluorochrome-labeled antibodies used were as follows: BV480/PE-MHC-II, FITC-CD86, APC-CD3, BV421/FITC-CD4, APC-CD11c, FITC-CCR7, PERCP/PE-CD8, APC-IFN-γ, AF647-Foxp3, APC-Cy7-CD45, BUV737-CD11b, BUV661-XCR1, PERCR/CY5.5-B220, RY610-CD172a, RB545-CD64. All of these antibodies were manufactured by BD Biosciences.

### Immunohistochemistry (IHC)

The tissues from patients were fixed with 10% (vol/vol) neutral-buffered formalin overnight and embedded in paraffin, and 5-µm-thick sections were prepared. Tissues sections were deparaffinized, followed by rehydrated in xvlene and graded concentrations of ethanol. Quenching of endogenous peroxidase activity was achieved using 3% hydrogen peroxide, while antigen retrieval was performed with an EDTA buffer. Then, sections were incubated overnight at 4°C in a humid chamber with the indicated primary antibodies. The 3,3'-diaminobenzidine detection system was employed to visualize the staining after 1 h incubation with secondary antibodies at room temperature. The mean optical density was employed to calculate the relative expression of CARD9 and YY1 using Image J software.

### Western blot (WB)

The extracted proteins were separated via sodium dodecyl sulfate polyacrylamide gel electrophoresis (SDS-PAGE), and the separated bands were transferred onto a polyvinyl difluoride membrane. Following blocking with 5% bovine serum albumin, the membrane was incubated with primary antibodies for 12 h at 4°C. The primary antibodies were specific for CARD9, YY1, Pan-Kla, HDAC2, Ubiquitin, BCL10, MALT1, p65, p-p65, SLC6A8, Histone H3, and GAPDH (Proteintech, Wuhan, China). The membrane was subsequently probed with appropriate secondary antibodies to visualize the protein bands. Anti-GAPDH antibody was used as a control.

### Hematoxylin-eosin (HE) staining

Cut appropriate amount of tumor tissue immersed in 4% paraformaldehyde for fixation. These specimens were embedded in paraffin and sectioned to slices, which were stained with HE.

### Immunofluorescence (IF)

Cells were planted on over slides and fixed with 4% paraformaldehyde for 30 min. After permeabilization using 0.5% Triton X-100 for 15 min, 5% BSA were utilized to block the nonspecific binding sites. The cells were incubated with primary antibodies overnight at 4°C. After being washed with PBST for 5 times, secondary antibody conjugated with Alexa Fluor-488 or -594 (Proteintech, Wuhan, China) was used for incubation. After 1 h, coverslips were washed with PBST without light and stained with 4',6-diamidino-2-phenylindole (DAPI). The images were captured using laser confocal microscope (Nikon).

### Co-immunoprecipitation (Co-IP)

For the co-immunoprecipitation assay (Co-IP), whole-cell extracts were lysed in IP lysis buffer (Beyotime) with protease inhibitor cocktail (Millipore) and PMSF (Beyotime). Cell lysates were centrifuged and cleared by incubation with 25 µL of Protein A/G magnetic beads or agarose (MedChemExpress) for 1.5 h at 4°C. The pre-cleared supernatant was subjected to IP using the indicated primary antibodies at 4°C overnight. The protein complexes were resolved by SDS-PAGE. Subsequently, western blot was performed.

### Lactate measurement

After preparation, the lactate concentration was measured in DCs cell supernatant using a lactate assay kit (Nanjing Jiancheng) according to the manufacturer's instructions. Cell samples were incubated at room temperature for 30 min, and enzyme-labeling devices were used to measure the absorbance at 570 nm.

### Panc02 cells co-cultured with DCs

DCs were placed in the lower layer of Trans-well culture in advance, and Panc02 cells were added to the upper layer of the chambers after DCs induction and differentiation were completed or other treatments were completed. After 24 h of co-culture, LPS and tumor antigens were given to stimulate DCs for 24 h, and then DCs were collected for subsequent processing.

### Panc02 cell supernatant processing

Panc02 cell culture 48 h supernatants were centrifuged (4000 r/min,10 min) to remove cellular debris and then placed in 3 kDa ultrafiltration centrifuge tubes and centrifuged (4°C, 5000 g, 40 min) to isolate protein (>3 kDa) and small molecule metabolite (<3 kDa) components. Subsequently, they were respectively added in a 1:1 ratio with the culture medium, and the effect on DCs was observed.

### SiRNA design and construction

SiRNA was synthesized with the assistance of Jin Kairui (Wuhan, China). The targeting sequences were described as follows: siYY1-1: CACAUCUUAACACACGCUAAA; siYY1-2: GUGGUUGAAGAGCAGAUCAUU; siYY1-3: GCCCUCAUAAAGGCUGCACAA; siLDHα-1: CGUCUCCCUGAAGUCUCUUAA; siLDHα-2: CGUGAACAUCUUCAAGUUCAU; siLDHα-3: GCAUCCCAUUUCCACCAUGAU; siP300-1: UAGGAGGUGAUAGUAUUCCGCTT; siP300-2: UUUAUCAAACUUAAUCCAGGGTT; siP300-3: AUACAAGGUGUCUCUAGUGUATT; siHDAC-1: AUUAUAUGGCAACUCAUUGGGTT; siHDAC2-2: AUUAUCUGGUCUUAUUGAUCGTT; siHDAC2-3: UUGCCGGUUUAAUUUCACAGCTT. Si-YY1, si-LDHα, si-P300, and si-HDAC2 were transfected in DCs using Hieff Trans Universal Transfection Reagent according to instructions.

### Plasmid construction and transfection

The OE-HDAC2 plasmid was biosynthesized by Weike Biology (Wuhan, China). For transfection, the cells were replaced with fresh culture medium 2 h prior to transfection, DNA was diluted to 2 µg per 100 µL of reduced serum medium. Then, Lipofectamine 3000 (Yeasen) was added to the dish and the mixture was incubated for 15 min at room temperature. Finally, 100 µL of mixture was added to each well of cells to be transfected, and was mixed gently. Cells were incubated for 36 h before further experiments.

### *In vitro* lactylation assay

The target protein YY1 (150 ng), p300 (30 ng), and lactyl-CoA (20 µM) were added to the reaction buffer and incubated at 30 °C for 2 h. The reaction buffer consisted of 50 mM HEPES, 30 mM KCl, 0.25 mM EDTA, 5 mM sodium butyrate, 2.5 mM DTT, and 5 mM MgCl_2_. Western blot analysis was subsequently performed to examine the protein expression of the reaction products.

### Chromatin immunoprecipitation assay

The ChIP assay was performed via SimpleChIP Plus Enzymatic Chromatin IP Kit (Cell Signaling Technology). BMDCs were crosslinked via formaldehyde after washing twice with PBS, and then lysed using sodium dodecyl sulfate buffer and sonicated. The ChIP dilution buffer was added to the fragmented chromatin, which was incubated with a rabbit antibody for 12 h. Rabbit IgG was used as a negative control, and rabbit anti-histone H3 antibody served as the positive control. After incubation with magnetic beads coated with protein A + G, collected immunoprecipitated products were eluted and digested with proteinase K to acquire DNA. The eluted DNA was subjected to ChIP-PCR or ChIP-qPCR with primers of indicated promoter region.

### DCs adoptive transfer experiment

DCs were given siRNA transfection first, and after 24 h of transfection, they were co-cultured with Panc02 cells for 24 h. Subsequently, DCs loaded with tumor antigens were obtained by giving LPS and tumor antigens stimulation for 12 h, and injected via tail vein into CD11c-DTR loaded tumor-bearing mice (5 × 10^6^ DCs). Due to the limited duration of maintenance of siRNA action and consideration of tail vein recovery in mice, injections were given twice weekly.

### Statistical analysis

The data were presented as mean ± standard deviation (mean ± SD), and GraphPad Prism 9 software was used to analyze the data and plots. Comparisons of data between two groups were made using Student's t-test, and comparisons of data between multiple groups were made using one-way analysis of variance (ANOVA). *P* < 0.05 means statistically significant (*P < 0.05, **P < 0.01, ***P < 0.001).

## Results

### Progressive loss of CARD9 and DC dysfunction in advancing PC

**Figure 1:**
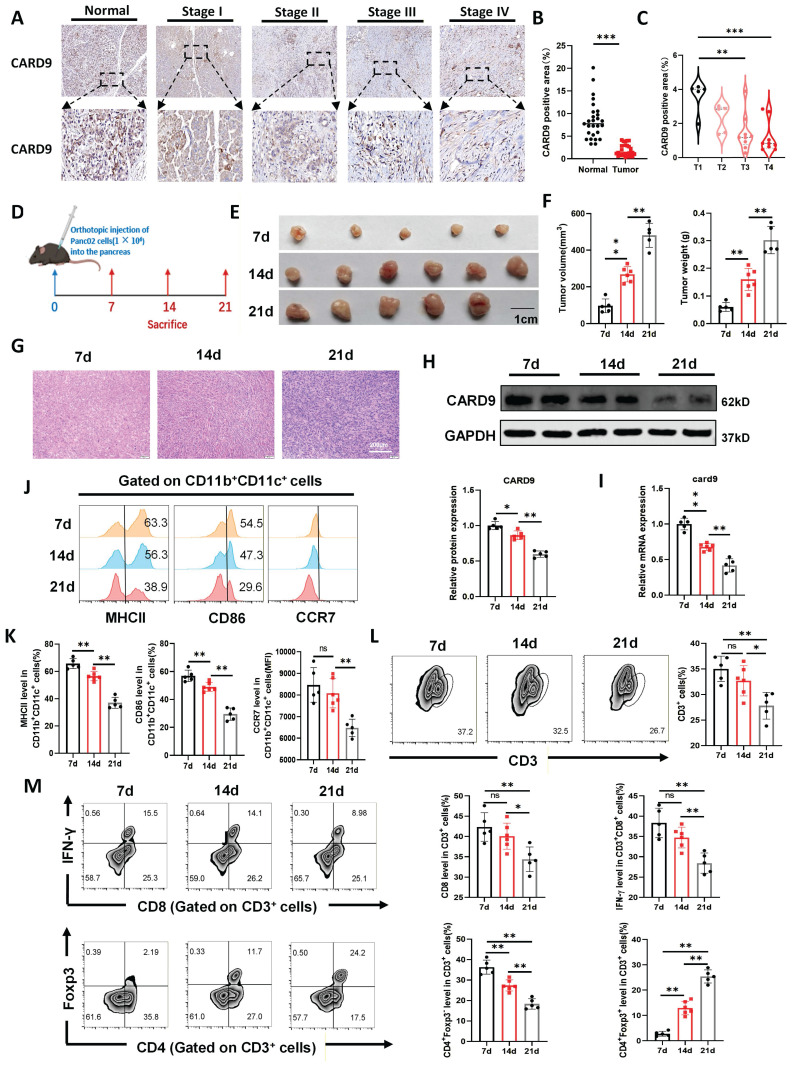
Progressive loss of CARD9 and DC dysfunction in advancing PC. A, B) The levels of CARD9 in PC and matched para-carcinoma tissues were visualized by IHC staining in tissue microarray. The representative picture of IHC staining and the visual field in the black square was enlarged down (n=27). C) CARD9 levels of AJCC stages T1 to T4 in PC patients. There are 5 PC patients in T1 stage, 5 in T2 stage, 8 in T3 stage, and 9 in T4 stage. D) A schematic diagram depicting the *in vivo* assessment of tumor by orthotopic injection of Panc02 cells (n=5/6). E, F) Tumor volume and weight of pancreatic cancer tumors were measured at 7, 14, and 21 days after implantation. G) Representative images using HE staining of tumor tissue sections. H, I) Western blot and qPCR were performed to detect the levels of CARD9 protein and gene expression in tumor tissues. J, K) CD86 and MHCII positivity as well as CCR7 levels in DCs were detected by flow cytometry. L) T-cell infiltration in tumor tissues was detected by flow cytometry. M) Proportion of CD8^+^ T cells among T cells and IFN-γ positivity among them were detected by flow cytometry. Proportion of Treg (CD4^+^Foxp3^+^) and non-Treg CD4^+^ T cells (CD4^+^Foxp3^-^) in tumor tissues were detected by flow cytometry. All data are presented as mean ± SD. Statistical analysis was performed by one-way ANOVA. *P < 0.05, **P < 0.01; ns, not significant.

To investigate the dynamic changes in CARD9 expression during PC progression, we assessed 27 tumor samples and matched para-carcinoma tissues by IHC staining. CARD9 levels were markedly reduced in tumor tissues and showed a clear decline with advancing disease stage (Fig. 1A-C). Subsequently, an orthotopic pancreatic cancer model was established in WT mice. Tumors were collected at 7, 14, and 21 days after implantation to evaluate growth, histological features, and CARD9 expression (Fig. 1D). As tumors progressed, histological analysis revealed increasingly abnormal nuclear morphology and reduced cytoplasm, indicating disease advancement (Fig. 1E-G). CARD9 expression progressively decreased at both the mRNA and protein levels, confirming its dynamic loss during PC progression (Fig. 1H, I).

Our previous studies demonstrated that CARD9 regulated PC progression primarily through modulation of DCs and T-cell responses. In addition, TIDE analysis revealed a positive correlation between CARD9 expression and the infiltration of DCs and CD8^+^ T cells in PC patients (Fig. S1A). Based on these observations, we next examined how immune cell populations changed during tumor development^10^. Flow cytometry analysis revealed a gradual decline in DC (CD11b^+^CD11c^+^) infiltration within the TME as tumor burden exacerbated (Fig. S1B). The expression of DC activation markers, including MHCII, CD86, and CCR7, also reduced over time, indicating mitigated DC activity (Fig. 1J, K). T-cell immunity showed a similar pattern. The proportions of total T cells (CD3^+^), CD8^+^ T cells (CD3^+^CD8^+^), and non-Treg CD4^+^ T cells (CD4^+^Foxp3^-^) steadily decreased during tumor progression, whereas Treg cells (CD4^+^Foxp3^+^) increased. Moreover, IFN-γ production by CD8^+^ T cells was reduced, reflecting impaired effector function (Fig. 1L, M). Together, these findings underscored that worsening PC was accompanied by a progressive loss of DC function and weakened T-cell immunity, alongside a pronounced increase in Treg infiltration. These changes suggested that PC may limit DC-mediated antitumor immunity by gradually reducing CARD9 expression in these cells.

### CARD9 is a key mediator of PC-induced DC suppression

**Figure 2:**
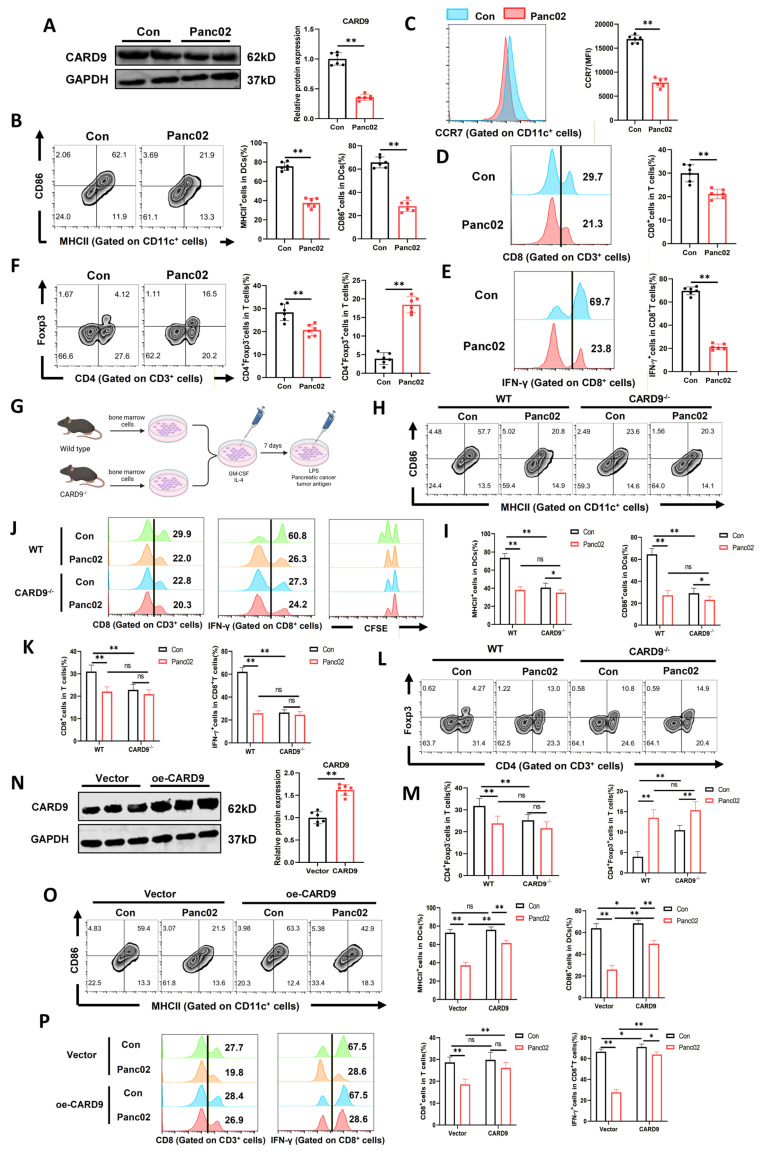
CARD9 is a key mediator of PC-induced DC suppression. A-C) DCs co-cultured with Panc02 cells for 24 h and then given LPS and tumor antigens stimulation for 24 h. A) The levels of CARD9 protein were detected in DCs by western blot. B, C) CD86 and MHCII positivity as well as CCR7 levels in DCs were detected by flow cytometry. D-F) DCs that had been cultured alone or co-cultured with Panc02 cells were incubated with T cells. D, E) Proportion of CD8^+^ T cells among T cells and IFN-γ positivity among them were detected by flow cytometry. F) Proportion of Treg (CD4^+^Foxp3^+^) and non-Treg CD4^+^ T cells (CD4^+^Foxp3^-^) in T cells were detected by flow cytometry. G) A schematic diagram depicting WT-DC and CARD9^-/-^-DC were cultured alone or co-cultured with Panc02 cells and subsequently given LPS and tumor antigens stimulation. H, I) CD86 and MHCII positivity in DCs were detected by flow cytometry. J-L) WT-DC and CARD9^-/-^-DC cultured alone or co-cultured with Panc02 cells were incubated with T cells after antigens stimulation, respectively. J, K) Proportion of CD8^+^ T cells among T cells and IFN-γ positivity among them were detected by flow cytometry and then CFSE was used to detect T-cell proliferative capacity. L, M) Proportion of Treg (CD4^+^Foxp3^+^) and non-Treg CD4^+^ T cells (CD4^+^Foxp3^-^) in T cells were detected by flow cytometry. N) The levels of CARD9 protein after DCs infected with CARD9 overexpressing adenovirus (MOI=200) were detected by western blot. O, P) WT-DC were infected with CARD9 overexpressing adenovirus and vector adenovirus, and both were cultured alone or co-cultured with Panc02 cells, followed by stimulation with LPS and tumor antigens. Then the above DCs were co-cultured with T cells for 24 h. CD86 and MHCII positivity as well as CCR7 levels in DCs were detected by flow cytometry. P) Proportion of CD8^+^ T cells among T cells and IFN-γ positivity among them were detected by flow cytometry. All data are presented as mean ± SD, n=6. Statistical analysis was performed using the Student *t* test or by two-way ANOVA. ^*^P < 0.05, ^**^P < 0.01; ns, not significant.

To verify the above hypothesis, PC cells (Panc02 or Aspc-1) were co-cultured with DCs and then stimulated with LPS and tumor antigens. In comparison with DCs cultured alone, co-cultured DCs showed a marked reduction in CARD9 mRNA and protein levels (Fig. 2A, S1C and S2A). Surface expression of the maturation markers, including MHCII, CD86, and CCR7, was also robustly decreased (Fig. 2B, C). To determine whether this effect was intrinsic to DCs rather than secondary to Panc02-derived cytokines, we measured cytokine transcripts (TNF-α, IL-12, IL-6, and IL-1β) in DCs and found that these were dramatically downregulated after co-culture (Fig. S1D). To functionally validate these findings, DCs were incubated with T cells. Exposure to Panc02 cells impaired the ability of DCs to expand CD8^+^ T cells and non-Treg CD4^+^ T cells. It also diminished IFN-γ production by CD8^+^ T cells and suppressed overall T-cell proliferation (Fig. 2D, E and S1E). In contrast, Panc02-exposed DCs promoted the differentiation of CD4^+^ T cells into Tregs (Fig. 2F).

To exclude potential direct effects of tumor cells on DCs through cell-cell contact, we next evaluated the impact of Panc02 cell-conditioned medium (CM) on DCs. DCs were treated with increasing concentrations of Panc02 CM (10%, 25%, or 50%). CARD9 expression descended in a concentration-dependent manner at both the mRNA and protein levels (Fig. S2B, C). Likewise, DC maturation markers (MHCII, CD86, and CCR7) and pro-inflammatory cytokines transcripts progressively declined with higher CM concentrations (Fig. S2D-F). Functionally, Panc02 CM reduced DC-driven expansion and activation of CD8^+^ T cells, alleviated overall T-cell proliferation and enhanced Treg differentiation in a concentration-dependent manner (Fig. S2G-J). The results we obtained above mirrored that both PC cells and their CM diminished CARD9 expression in DCs, impaired their maturation and weakened their ability to activate T cells.

To further determine whether Panc02-induced DC suppression occurs primarily through CARD9 downregulation, bone marrow cells from WT and CARD9^-/-^ mice were differentiated into DCs. These cells were either cultured alone or co-cultured with Panc02 cells prior to stimulation (Fig. 2G, S3A). Consistently, Panc02 cells suppressed the expression of maturation markers (MHCII, CD86, and CCR7) in WT-DCs. They also reduced pro-inflammatory cytokines transcription (TNF-α, IL-12, IL-6, and IL-1β) and impeded the capacity of WT-DCs to activate and expand T cells (Fig. 2H-M, S3B-C). While Panc02 cells also decreased maturation markers in CARD9^-/-^-DCs, these effects were notably weaker. Meanwhile, CARD9^-/-^-DCs showed no significant discrepancy in CCR7, cytokine expression, CD8^+^ T cell activation and proliferation when exposed to Panc02 (Fig. 2H-M, S3B-C).

Subsequently, we explored whether restoring CARD9 expression could counteract PC cell-mediated suppression. DCs were infected with control (vector) or CARD9-overexpressing adenovirus (MOI=200) and then co-cultured with Panc02 cells (Fig. 2N, S3D). CARD9 overexpression considerably upregulated DC maturation marker expression, in some cases exceeding levels in DCs cultured alone (Fig. 2O, S3E).

It also enhanced cytokine production and improved T-cell activation (Fig. S3F). In the presence of Panc02 cells, CARD9 restoration obviously promoted CD8^+^ T cell activation, increased IFN-γ secretion, enhanced T-cell proliferation, while suppressing Treg differentiation (Fig. 2P, S3G-H). Together, the aforementioned findings indicated that CARD9 deficiency is a principal mechanism by which PC cells suppressed DC maturation and function. Finally, to identify which DC subset was primarily affected by CARD9 deficiency, we established orthotopic pancreatic cancer models in WT and CARD9^-/-^ mice. Tumor tissues were analyzed by dimensionality reduction based on DC subset markers (CD64 for moDCs, CD172a for cDC2, B220 for pDCs, and XCR1 for cDC1). Notably, the cDC1 population was markedly reduced in CARD9^-/-^ mice compared with WT controls, whereas the distribution of moDCs, cDC2, and pDCs showed minor differences (Fig. S4A, S4B). These results indicated that CARD9 deficiency primarily impaired the cDC1 subset rather than other DC populations.

### YY1 directly binds to CARD9 and suppresses its activation in DCs

**Figure 3:**
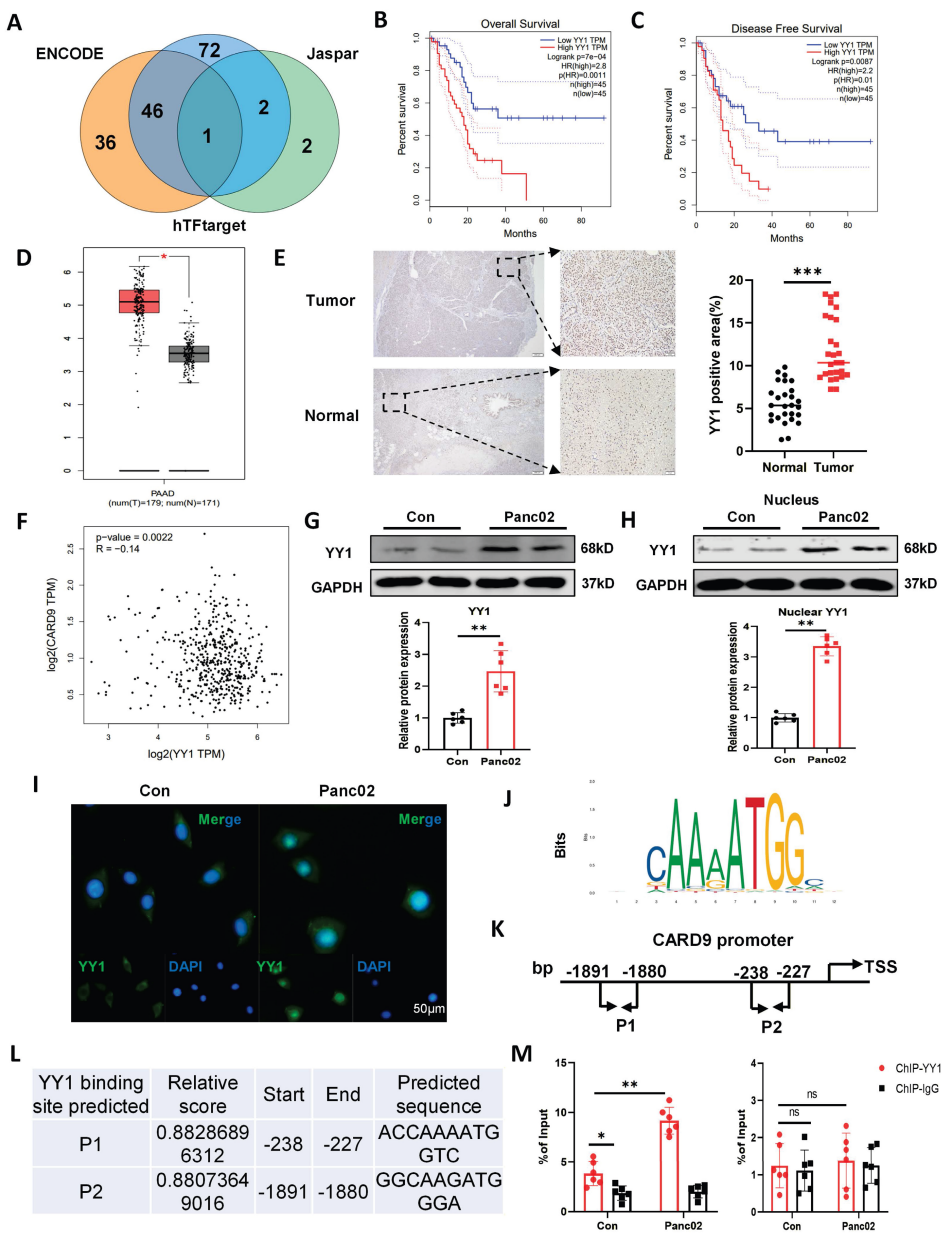
YY1 directly binds to CARD9 and suppresses its activation in DCs. A) Three databases, ENCODE, Jaspar, and hTFtarget, predicted potential transcription factors for CARD9. B, C) YY1 expression levels in tumor tissues were analyzed using the GEPIA database in relation to overall survival and disease-free survival of PC patients. D) Differences in YY1 expression between tumor tissues and normal pancreatic tissues of PC patients were analyzed by GEPIA database. E) The levels of YY1 in PC and matched para-carcinoma tissues were visualized by IHC staining in tissue microarray. The representative picture of IHC staining and the visual field in the black square was enlarged right (n=27). F) Correlation of CARD9 and YY1 expression in tumor tissues of PC patients was analyzed by GEPIA database. G, H) The levels of total YY1 and intranuclear protein were detected by western blot in DCs after alone or co-cultured with Panc02 cells. I) Distribution of YY1 inside and outside the DCs nucleus was observed by immunofluorescence of control or co-culture with Panc02 cells. J-L) YY1 binding sites on the CARD9 promoter were predicted by Jaspar database. M) ChIP-qPCR experiments were performed to analyze the binding of YY1 to the CARD9 promoter in DCs cultured alone or co-cultured with Panc02 cells. All data are presented as mean ± SD, n=6. Statistical analysis was performed using the Student *t* test or by two-way ANOVA. ^*^P < 0.05, ^**^P < 0.01; ns, not significant.

As noted above, Panc02 cells suppressed DCs maturation and function by reducing CARD9 expression, prompting further investigation into the underlying mechanisms. Our prior data showed that Panc02 cells lowered CARD9 transcript levels in DCs, suggesting a crucial involvement of transcriptional regulation. We performed in silico analyses using ENCODE, Jaspar, and hTFtarget databases to identify potential transcription factors regulating CARD9. By intersecting the results from these approaches, YY1 was the only candidate consistently predicted across all three databases (Fig. 3A). Meanwhile, GEPIA analysis further showed that high YY1 expression was associated with poorer overall and disease-free survival. YY1 was also highly expressed in tumor samples from both public databases and our clinical cohort (Fig. 3B-E). In addition, YY1 was also inversely correlated with CARD9 expression in PC datasets, suggesting regulatory interplay (Fig. 3F). Together with the observed inverse pattern of CARD9 expression during disease progression, these findings support the hypothesis that YY1 negatively regulated CARD9.

To verify this hypothesis, we examined YY1 expression and activation in DCs cultured alone or with Panc02 cells. Co-culture system substantially enhanced YY1 protein levels and triggered its nuclear localization, indicating activation of YY1 in DCs exposed to Panc02 cells (Fig. 3G-I). We then assessed whether YY1 directly regulated CARD9 transcription. Bioinformatic analysis identified two putative YY1-binding sites within the CARD9 promoter region (Fig. 3J-L). ChIP-qPCR experiment reinforced that Panc02 co-culture notably strengthened YY1 binding to the CARD9 promoter, specifically at the consensus site “ACCAAAATGGTC” (Fig. 3M). These results delineated that YY1 directly bound to the CARD9 promoter and acted as a transcriptional repressor, contributing to CARD9 downregulation in DCs exposed to Panc02 cells.

### YY1 knockdown restores CARD9 and reverses PC-induced DC dysfunction

**Figure 4:**
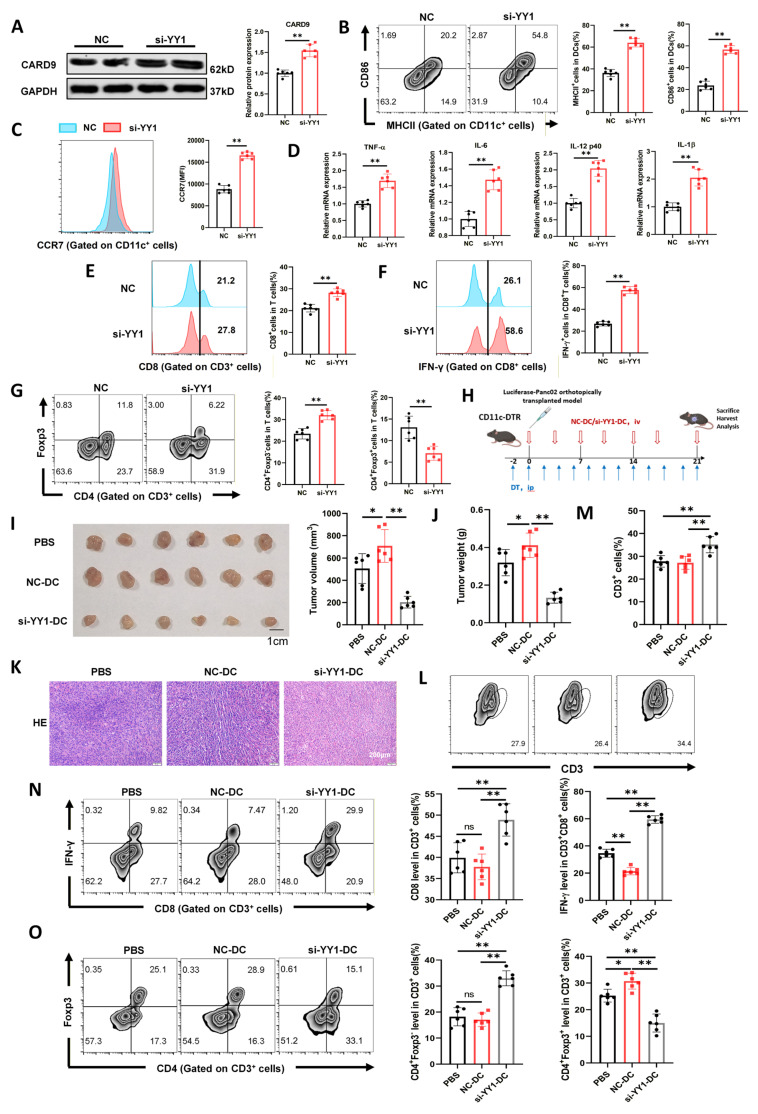
YY1 knockdown restores CARD9 and reverses PC-induced DC dysfunction. A-D) The siRNAs constructed to interfere with YY1 expression and the negative control (NC) were transfected into DCs, which were subsequently co-cultured with Panc02 cells for 24 h. A) Effect of si-YY1 on CARD9 protein levels in DCs was detected by western blot. B, C) CD86 and MHCII positivity as well as CCR7 levels in DCs were detected by flow cytometry. D) Transcript levels of inflammatory factors in DCs were detected by qPCR. E-H) DCs transfected with NC and si-YY1 were co-cultured with Panc02 cells and given antigenic stimulation, followed by co-culture with T cells. E, F) Proportion of CD8^+^ T cells among T cells and IFN-γ positivity among them were detected by flow cytometry. G) Proportion of Treg (CD4^+^Foxp3^+^) and non-Treg CD4^+^ T cells (CD4^+^Foxp3^-^) in T cells were detected by flow cytometry. H) A schematic diagram depicting the in vivo assessment of tumor by DCs adoptive transfer experiments (n=6). I, J) Pancreatic cancer tumor volume and weight were measured at 21 days after implantation. K) Tumor tissue lesions in mice were observed using HE staining. L, M) T-cell infiltration in tumor tissues was detected by flow cytometry. N) Proportion of CD8^+^ T cells among T cells and IFN-γ positivity among them were detected by flow cytometry. O) Proportion of Treg (CD4^+^Foxp3^+^) and non-Treg CD4^+^ T cells (CD4^+^Foxp3^-^) in tumor tissues were detected by flow cytometry. All data are presented as mean ± SD, n=6. Statistical analysis was performed using the Student *t* test or by one-way ANOVA. ^*^P < 0.05, ^**^P < 0.01; ns, not significant.

To delve deeper into the biological role of YY1, we silenced YY1 in DCs (si-YY1) using specific interfering RNAs (siRNAs) and evaluated its effects on CARD9 expression, DC maturation and downstream immune functions (Fig. S4C, S4D). Following co-culture with Panc02 cells and stimulation, YY1 knockdown restored CARD9 at both the mRNA and protein levels (Fig. 4A, S4E). Flow cytometry further illustrated that si-YY1 DCs displayed higher expression of the maturation markers MHCII, CD86, and CCR7 (Fig. 4B, C). Consistent with this, transcripts of pro-inflammatory cytokines (TNF-α, IL-12, IL-6, and IL-1β) were also elevated in the si-YY1 group (Fig. 4D). To assess functional consequences, these DCs were then incubated with T cells. YY1 knockdown enhanced DC-mediated activation of CD8^+^ T cells and increased IFN-γ production. It also facilitated overall T-cell proliferation while reducing the differentiation of CD4^+^ T cells into Tregs (Fig. 4E-G, S4F).

To validate these observations *in vivo*, we conducted orthotopic PC models in CD11c-DTR mice. Mice received intravenous injections of PBS, control DCs (NC-DCs), or YY1-silenced DCs (si-YY1-DCs) twice weekly (Fig. 4H). Prior to transfer, both DC populations were co-cultured with Panc02 cells and stimulated to ensure functional activation. After 21 days, tumor burden was evaluated. Compared with PBS treatment, NC-DC administration accelerated tumor growth, whereas si-YY1-DC treatment ameliorated this effect (Fig. 4I, J). H&E staining further showed reduced nuclear enlargement and chromatin condensation in tumors from the si-YY1-DC group, indicating alleviated tumor pathology (Fig. 4K).

We next examined T-cell infiltration and activity within the tumor microenvironment. NC-DC treatment did not appreciably alter the number of total CD3^+^ T cells, CD8^+^ T cells, or non-Treg CD4^+^ T cells compared with PBS, but attenuated IFN-γ-producing CD8^+^ T cells and increased Treg frequencies. Conversely, si-YY1-DC treatment dramatically enhanced overall T-cell infiltration, including CD8^+^ T cells and non-Treg CD4^+^ T cells. It also advanced IFN-γ production by CD8^+^ T cells and significantly downregulated Treg proportions relative to both PBS and NC-DC groups (Fig. 4L-O). Collectively, silencing YY1 expression in DCs restored CARD9 expression and DC functional competence, thereby potentiating T-cell-mediated antitumor immunity and alleviating PC progression *in vivo*.

### Tumor-derived lactate drives YY1 activation and CARD9 suppression in DCs

**Figure 5:**
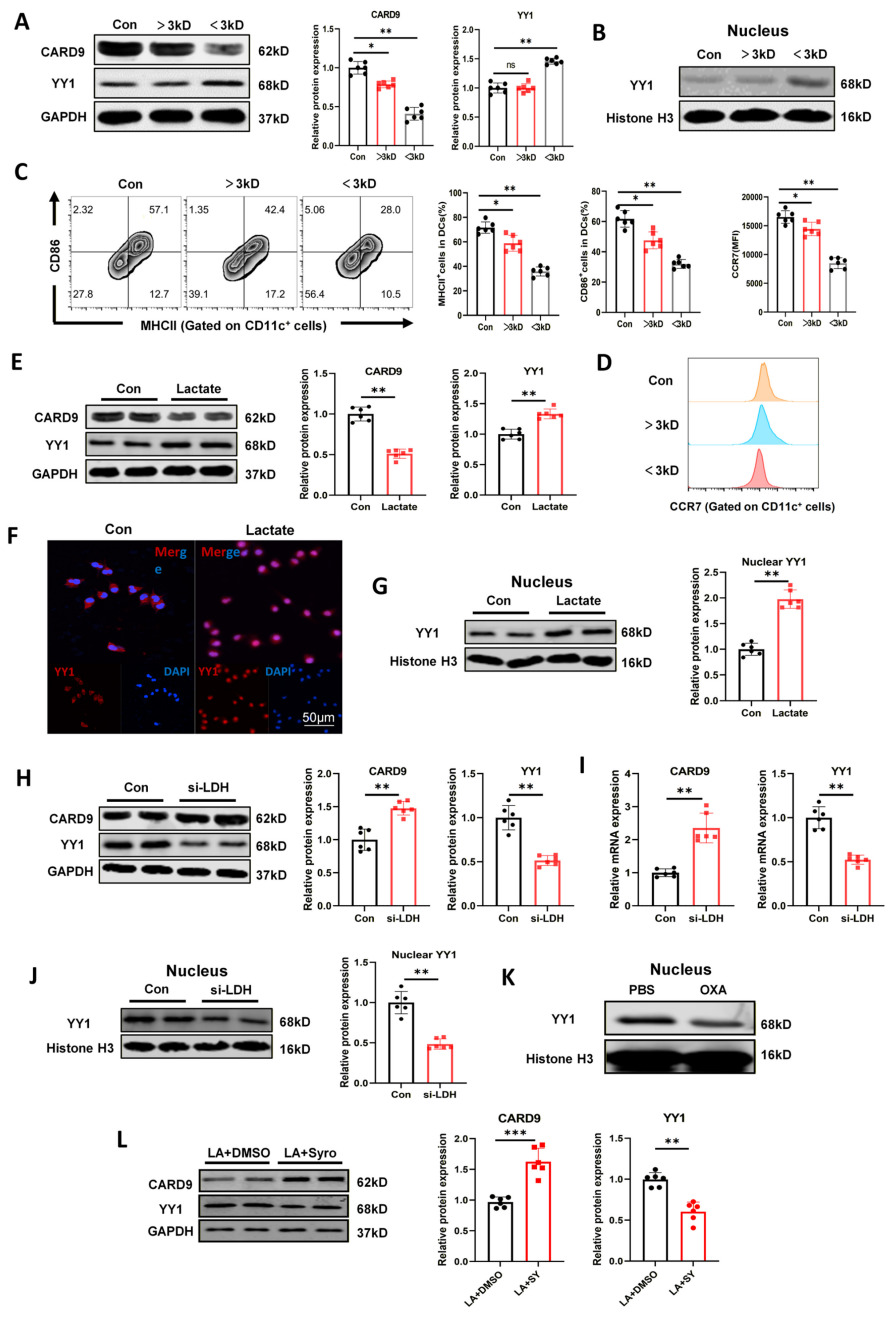
Tumor derived lactate drives YY1 activation and CARD9 suppression in DCs. A, B) Different components of the supernatant from Panc02 were separated using 3 kDa ultrafiltration centrifuge tubes, and the levels of CARD9 and YY1 total proteins and intranuclear YY1 proteins were detected in DCs by western blot after co-culturing with DCs for 24 h. C, D) Effects of different components of Panc02 supernatant on CD86, MHCII positivity and CCR7 levels in DCs were detected by flow cytometry. E, G) After exogenous addition of lactate (15 mM) for 24 h, the levels of CARD9 and YY1 total protein and intranuclear YY1 proteins were detected in DCs by western blot. F) Distribution of YY1 inside and outside the DCs nucleus after exogenous addition of lactate was observed by immunofluorescence. H-J) Negative control and si-LDHα were transfected into Panc02 cells, and these Panc02 cells were subsequently co-cultured with DCs. Western blot and qPCR were performed to detect the levels of total CARD9, YY1 protein and intranuclear YY1 proteins in DCs. K) Oxamate (OXA, 20 mM) or PBS were treated DCs for 24 h, the levels of YY1 protein were detected in the nuclei of cells by western blot. L) The levels of CARD9 and YY1 protein were detected in DCs by western blot after treatment with the dual MCT1/MCT4 inhibitor Syrosingopine (Syro, 15 µM) or DMSO for 24 h in the presence of lactate. All data are presented as mean ± SD, n=6. Statistical analysis was performed using the Student *t* test or by one-way ANOVA. ^*^P < 0.05, ^**^P < 0.01; ns, not significant.

The above results elucidated that both direct co-culture with Panc02 cells and treatment with its cell supernatants suppressed DC maturation and reduced CARD9 expression, suggesting that tumor-derived soluble factors may contribute to this effect. To identify the specific components, Panc02 supernatants were separated into protein (>3 kDa) and small-molecule (<3 kDa) fractions. Each fraction was then applied to DCs. Both fractions inhibited CARD9 expression, yet the small-molecule metabolites exerted a prominently stronger effect (Fig. 5A, S5A). Notably, only the small-molecule fraction upregulated total and nuclear YY1 levels in DCs, whereas the protein fraction showed no such effect (Fig. 5A-B, S5A-B). Additionally, both fractions mitigated the surface expression of DC maturation markers MHCII, CD86, and CCR7, with a more pronounced decline in cells exposed to the small-molecule metabolites (Fig. 5C, D). When these DCs were co-cultured with T cells, the small-molecule fraction impaired CD8^+^ T cell proliferation, while the protein fraction produced no detectable change (Fig. S5C). Both fractions attenuated CD8^+^ T cell activation, overall T-cell proliferation and facilitated Treg differentiation. However, the suppressive effects were consistently stronger in the small-molecule group (Fig. S5D-F).

These findings explicated that small-molecule metabolites played a pivotal role in CARD9 suppression and YY1 activation in DCs. Non-targeted metabolomics of serum samples from PDAC patients and healthy groups have identified lactate as an elevated metabolite, and previous studies also reported that lactate could impair the function of several immune cell types, including DCs^24^. Building on this foundation, we hypothesized that PC cells may regulate YY1 activation and CARD9 expression in DCs through lactate secretion. Lactate release was quantified in multiple PC cell lines at 24 h and 48 h. Among them, Panc02 cells exhibited the highest lactate production at 48 h. Therefore, Panc02 cells were selected as the *in vitro* model for subsequent mechanistic investigations (Fig. S5G). Primary DCs were stimulated with LPS and tumor antigens following lactate treatment. Lactate strikingly reduced CARD9 expression at both the mRNA and protein levels, while simultaneously increasing YY1 expression and promoting its nuclear translocation (Fig. 5E-G, S6A). These results elucidated that exogenous lactate suppressed CARD9 while sharply inducing YY1 activation in DCs.

Lactate dehydrogenase (LDH-α) catalyzes the conversion of pyruvate to lactate and is a major driver in lactate production^25^. To validate whether lactate derived from Panc02 cells contributes to DC suppression, LDH-α expression was silenced in Panc02 cells using siRNAs. Reduced lactate production was verified by qPCR and lactate release assays (Fig. S6B, C). When DCs were co-cultured with si-LDHα Panc02 cells, CARD9 expression was evidently restored at both the mRNA and protein levels. This was accompanied by reduced YY1 expression and diminished nuclear accumulation, in contrast to DCs exposed to control Panc02 cells (Fig. 5H-J). Similarly, pretreatment of Panc02 cells with LDH-α inhibitor oxamate (OXA), which abrogated endogenous lactate production, resulted in reducing nuclear YY1 levels in DCs after co-culture (Fig. 5K). Since lactate transport was primarily dependent on monocarboxylate transporters (MCTs), we next blocked lactate uptake using the dual MCT-1/MCT-4 inhibitor syrosingopine (Syro). Syro treatment decreased YY1 and increased CARD9 expression relative to DMSO-treated controls following lactate stimulation (Fig. 5L). Collectively, lactate was a key metabolite through which Panc02 cells promoted YY1 activation, suppressed CARD9 expression and impaired DC function.

### Lactate-induced YY1 lactylation is regulated by writers p300 and erasers HDAC2

**Figure 6:**
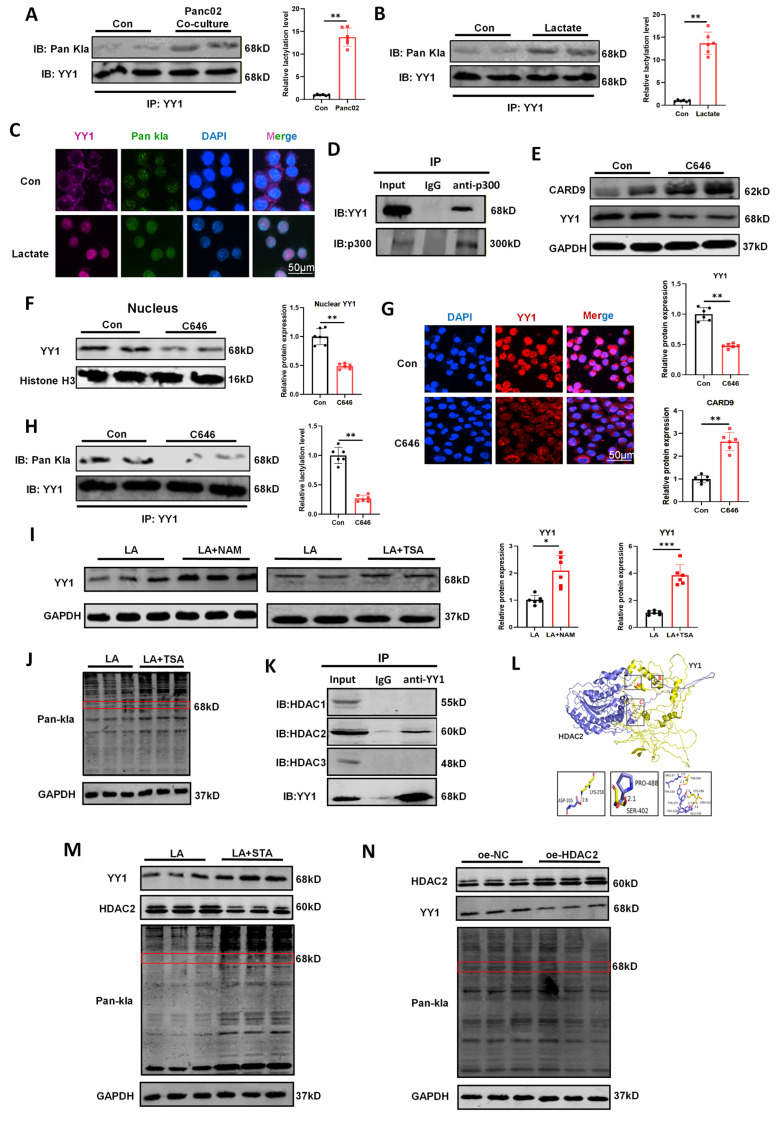
Lactate-induced YY1 lactylation is regulated by writers p300 and erasers HDAC2. A) The effect of Panc02 cells on the level of YY1 lactylation was detected by Co-IP assay in DCs. B) The effect of lactate on the level of YY1 lactylation was detected by Co-IP assay in DCs. C) Distribution of YY1 and pan-Kla in DCs under lactate treatment was observed by immunofluorescence. D) The interaction between p300 and YY1 was detected by Co-IP assay in DCs. E, F) The levels of CARD9 and YY1 total protein and intranuclear YY1 protein were detected in DCs by western blot after treatment with the p300 inhibitor C646 (100 µM) for 24 h in the presence of lactate. G) Distribution of YY1 inside and outside the DCs nucleus under C646 treatment was observed by immunofluorescence. H) The effect of C646 on the level of YY1 lactylation was detected by Co-IP assay in DCs. I, J) The levels of YY1 protein and lactylation after treatment with either SIRT inhibitor Nicotinamide (NAM, 10 mM) or HDAC inhibitor Trichostatin A (TSA, 500 nM) for 24 h in the presence of lactate were detected in DCs by western blot. K) The interaction between these erasers (histone deacetylase 1-3) and YY1 was detected by Co-IP assay in DCs. L) Molecular docking diagram of YY1 and HDAC2. M) The levels of YY1, HDAC2 protein and lactylation after treatment with HDAC2 inhibitor Santacruzamate A (STA, 20 µM) for 24 h in the presence of lactate were detected in DCs by western blot. N) The levels of YY1, HDAC2 protein and lactylation in HDAC2 overexpressing DC2.4 cells in the presence of lactate were detected in DCs by western blot. All data are presented as mean ± SD, n=3/6. Statistical analysis was performed using the Student *t* test or by one-way ANOVA. ^*^P < 0.05, ^**^P < 0.01; ns, not significant.

Based on current research, lactate appeared to be essential for YY1 activation and the subsequent downregulation of CARD9. A large number of studies have reported that lactylation, a lactate-derived PTM, covalently modifies lysine residues and could alter substrate conformation or protein-protein interactions^26^. Hence, we speculated that lactate activated YY1 by inducing its lactylation, leading to restricting CARD9 transcription. To test this, we first assessed YY1 lactylation levels by IP assays. YY1 lactylation was remarkably elevated in DCs co-cultured with Panc02 cells versus cultured alone (Fig. 6A). Lactate treatment similarly enhanced YY1 lactylation and promoted its nuclear translocation, supporting our proposed mechanism (Fig. 6B, C). Given that p300 is a well-characterized acetyltransferase and is known to regulate several proteins, including YY1. We substantially examined whether p300 participated in YY1 lactylation in DCs^27^. Co-IP assays confirmed the endogenous interaction between YY1 and p300 in DCs (Fig. 6D). DCs were then treated with lactate in the presence of either the p300 inhibitor C646 (C646 group) or DMSO (Con group) as a control. C646 group sharply enhanced CARD9 expression, reduced both total and nuclear YY1 levels, and suppressed YY1 lactylation compared with Con group (Fig. 6E-H, S6D). Furthermore, we designed three siRNA sequences targeting p300 and evaluated their knockdown efficiency using qPCR. Among them, sequence 1 showed the highest efficiency and was used in subsequent experiments (Fig. S6E). Then under the same lactate stimulation conditions, p300 knockdown markedly reduced YY1 lactylation and prevented YY1 activation (Fig. S6F). To investigate whether p300 could transfer a lactyl group to YY1, we performed an *in vitro* lactylation assay by incubating purified YY1 with p300 in a reaction system containing lactyl-CoA. Strong lactylation of YY1 was observed only in the presence of both p300 and lactyl-CoA, whereas heat-inactivated p300 failed to induce YY1 lactylation (Fig. S6G). These findings reinforced that p300 functioned as the lactylation “writers” for YY1, mediating its lactate-dependent modification and activation.

In addition to writer-mediated protein lactylation, this modification can also be catalyzed by erasers, including sirtuin 1-3 (SIRT1-3) and histone deacetylase 1-3 (HDAC1-3)^28^. We sought to investigate which enzyme was of paramount importance for YY1 delactylation. DCs were treated with the HDAC inhibitor Trichostatin A (TSA) or the SIRT inhibitor Nicotinamide (NAM). Although both inhibitors elevated YY1 protein levels, only TSA enhanced its lactylation status, suggesting an HDAC-dependent mechanism (Fig. 6.I-J, S6H). To further pinpoint the critical eraser for YY1 delactylation, we performed Co-IP assays to examine interactions between YY1 and HDAC1-3. Among the tested enzymes, an evident interaction was detected between YY1 and HDAC2 (Fig. 6K). Molecular docking analysis further verified the reliability of the above results (http://hdock.phys.hust.edu.cn), revealing stable binding between YY1 and HDAC2 mediated by multiple hydrogen bonds (Fig. 6L). Consistent with these results, treatment with Santacruzamate A (STA), a selective HDAC2 inhibitor, increased both YY1 expression and its lactylation levels (Fig. 6M). Similarly, HDAC2 knockdown using siRNA significantly elevated YY1 lactylation and enhanced YY1 activation (Fig. S7A, S7B). Conversely, we overexpressed HDAC2 in DC2.4 using plasmids, which proved that enhancing HDAC2 expression could significantly alleviate lactate-induced YY1 expression and lactylation (Fig. 6N). In summary, we deduced that HDAC2 functions as the key “eraser” regulating YY1 delactylation in DCs, complementing the role of p300 as the corresponding “writer” and establishing a reversible mechanism controlling YY1 activation.

### YY1-K183 lactylation enhances YY1 transcriptional activity and stability

**Figure 7:**
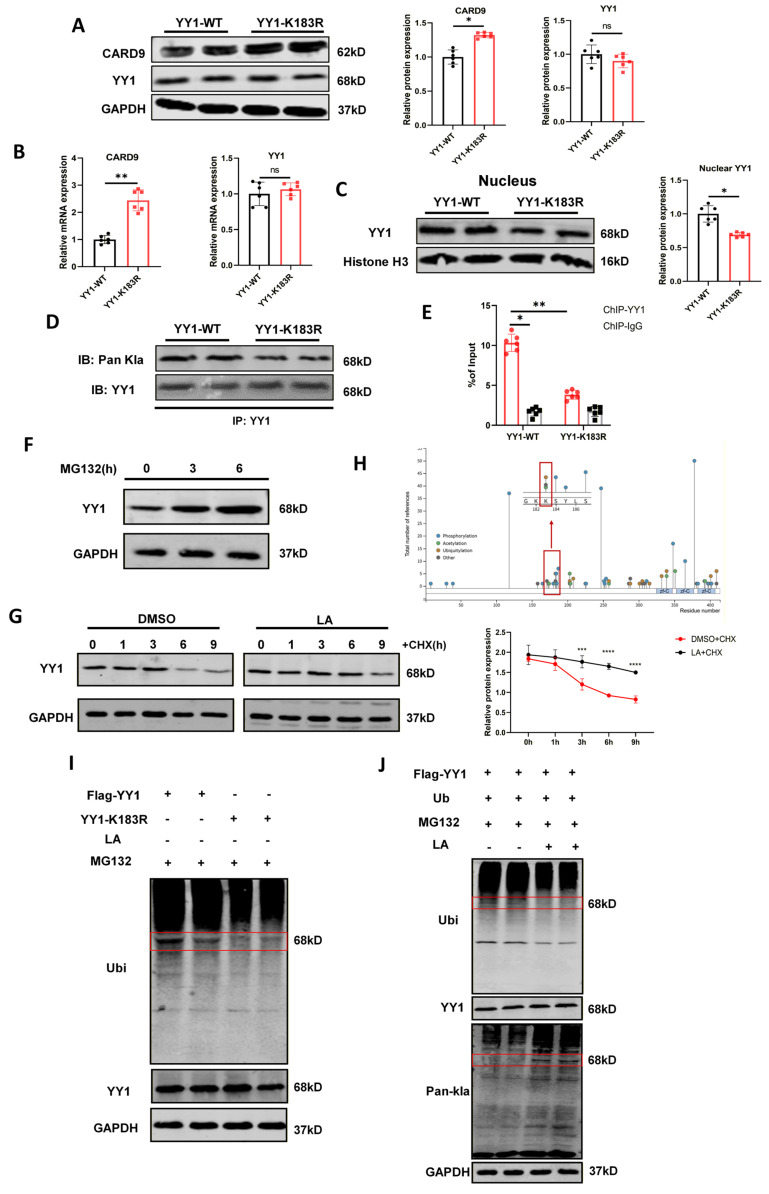
YY1-K183 lactylation enhances YY1 transcriptional activity and stability. A-C) Normal YY1 (YY1-WT) and K183R mutated YY1 (YY1-K183R, lysine residues to be mutated to arginine) plasmids were transfected into DC2.4 for 36 h, and the cells were treated with lactate at 12 h after transfection. Western blot and qPCR were performed to detect the levels of total CARD9, YY1 protein and intranuclear YY1 proteins in DC2.4 after transfection. D) The effect of the K183R mutation on the level of YY1 lactylation was detected by Co-IP assay in DC2.4. E) The inhibition of YY1 binding to the CARD9 promoter in DC2.4 by the K183R mutation was detected by ChIP-qPCR assay. F) The levels of YY1 protein in DCs treated with 10 µM MG132 for indicated time were detected by western blot. G) The levels of YY1 protein in DCs treated with or without 15 mM lactate and along with 0.1 µg/mL cycloheximide (CHX) for the indicated time were detected by western blot. H) Overview of YY1 post-translational modification sites shown from the PhosphoSitePlus database (https://www.phosphosite.org). I) DC2.4 cells were co-transfected with Flag-YY1, YY1-K183R, and Vector-YY1. Cells were treated with 10 µM MG132 for 8 h and detected YY1 and pan-ubiquitination levels by western blot. J) DC2.4 cells were transfected with Flag-YY1. Cells were treated with lactate and ubiquitination for 24 h and MG132 for 8 h. The levels of YY1 protein, lactylation and pan-ubiquitination were detected by western blot. All data are presented as mean ± SD, n=3/6. Statistical analysis was performed using the Student *t* test or by two-way ANOVA. ^*^P < 0.05, ^**^P < 0.01; ns, not significant.

Protein lactylation typically occurs on lysine residues of substrate proteins, and previous proteomic analyses have identified lysine 183 (K183) as a potential lactylation site on YY1^29^. To validate this, DC2.4 cells were transfected with plasmids carrying YY1-WT or YY1-K183R mutant. After lactate treatment, the K183R mutation did not alter total YY1 expression. However, it increased CARD9 expression and mitigated YY1 lactylation. In addition, the mutation diminished nuclear YY1 accumulation and weakened its binding to the CARD9 promoter in comparison to normal YY1 (Fig. 7A-E, S7C). We further examined the effects of this mutation on the CBM-p65-SLC6A8-creatine axis and DC maturation, linking these observations to our previous research. The results showed that YY1 with K183 site mutation failed to suppress CBM complex activation, p65 phosphorylation, and SLC6A8 expression (Fig. S7D). Then, flow cytometry analysis revealed that inhibition of YY1 lactylation significantly promoted the expression of the maturation markers CD86, MHCII, and CCR7 on DCs (Fig. S7E, S7F). Increasing evidence implicated that lactate could directly promote protein lactylation to stabilize its expression. Consistent with this, YY1 degradation in DCs predominantly occurred through the proteasome pathway, as shown by YY1 accumulation following MG132 treatment (Fig. 7F). To further assess YY1 stability, we treated cells with cycloheximide (CHX) to block new protein synthesis in the presence or absence of lactate. Lactate treatment prolonged the half-life of YY1, indicating that lactate contributes to YY1 stabilization (Fig. 7G). Database analysis using PhosphoSitePlus revealed that the K183 site of YY1 was also a potential ubiquitylation site (Fig. 7H). Supporting this prediction, we transfected YY1-WT and YY1-K183R plasmids into DC2.4 cells without lactate.

The K183R mutation effectively reduced YY1 ubiquitination level (Fig. 7I). Current studies have suggested that protein lactylation can impact their own or other proteins' ubiquitination modifications, thereby influencing protein stability and degradation. To determine whether YY1 lactylation affected its ubiquitination, we explored the global expression of lactylation and ubiquitination levels in DCs. Interestingly, lactate treatment increased global lactylation while concurrently decreasing global ubiquitination (Fig. 7J). Overall, these findings identified K183 as the major lactylation site on YY1 in DCs and reinforced that lactate-induced lactylation enhanced YY1 stability by limiting its ubiquitination, thereby strengthening its regulatory effects on CARD9 transcription.

## Discussion

PC remains one of the most lethal malignancies, characterized by dismal clinical outcomes and poor patient prognosis^30^. It is projected to become the second leading cause of cancer-related mortality by 2030, underscoring the urgent need for novel and effective therapeutic strategies^2^. Several approaches have been explored to improve the responsiveness of PC to immune checkpoint inhibitors and combination regimens. However, their efficacy remains severely constrained by the highly immunosuppressive tumor environment and the extensive genetic heterogeneity arising from diverse oncogenic mutations 31-33. In this study, we observed that reduced CARD9 expression and progressive DC dysfunction in pancreatic tumors were associated with advancing PC. Mechanistically, we identified a tumor-driven pathway in which PC cells released lactate that was subsequently taken up by DCs, leading to YY1 lactylation and nuclear translocation. Activated YY1 directly bound to the CARD9 promoter and repressed its transcription, resulting in impairing DC maturation and function. Consequently, the lactate-YY1-CARD9 axis weakened antitumor immunity and facilitated immune evasion by PC cells.

Conventional dendritic cells (cDCs) are pivotal initiators of antigen presentation and T-cell priming^34^. Upon activation, DCs upregulate a broad repertoire of molecules essential for immune activation and migration, including chemokines, pattern recognition receptors, costimulatory receptors (for example, CD80, CD86, and CD40), MHC class I and II, and pro-inflammatory cytokines (for example, IL-12, IL-1β, and TNF). However, DCs are scarce and functionally impaired due to the pronounced immunosuppressive conditions within the tumor microenvironment^35^. CARD9, which is predominantly expressed in DCs and macrophages, functions as a central adaptor molecule that integrates innate and adaptive immune signaling^36^. Our prior study demonstrated that CARD9 deficiency disrupted CBM (CARD9-BCL10-MALT1) complex formation and inhibited the p65-SLC6A8 axis, leading to impaired creatine uptake in DCs. Creatine shortage culminated in immature and dysfunctional DCs, which failed to present neoantigens to CD8^+^ T cells. This dysregulation blunted the cytotoxic activity of CD8^+^ T cells against PC and aggravated tumor progression^10^. Yet, the mechanisms driving CARD9 downregulation in DCs remained largely unexplored. Here, analyses of patient samples and *in vivo* models revealed that reduced CARD9 expression correlated with advanced clinicopathological stages and DC dysfunction in PC. Supporting this idea, co-culture of DCs with Panc02 cells or their conditioned medium considerably attenuated DC maturation, cytokine production and CARD9 expression. In addition, experiments using CARD9^-/-^ mice and CARD9-overexpressing DCs implied that inhibitory effects of PC cells on DC maturation and T-cell activation were largely mediated through CARD9 downregulation.

Although CARD9 deficiency is a crucial contributor to PC-induced impairment of DC function, the upstream mechanisms governing CARD9 expression prompted further investigation. YY1, a transcription factor capable of functioning as both an activator and repressor, emerged as a potential candidate regulator of CARD9 based on predictions from three databases. YY1 is a ubiquitous factor renowned for its flexibility and critical role in several physiological processes^37^. Recent studies have implicated YY1 in PC biology. For instance, Chen *et al*. found a potential therapeutic strategy targeting FER/STAT3/YY1/MMP2 axis in PDAC and outlined a mechanism whereby YY1 inhibits PDAC cell migration and invasion by downregulating the expression of FER^38^. Consistent with these reports, our bioinformatics and clinical analyses revealed that YY1 expression was elevated in pancreatic tumors and positively correlated with disease progression and poor prognosis, suggesting a potential inhibitory role in regulating CARD9. ChIP-qPCR experiments confirmed that YY1 directly bound to the CARD9 promoter in the nucleus. Furthermore, functional studies using YY1 knockdown *in vitro* and adoptive transfer of si-YY1-DCs validated that YY1 was a key transcription repressor through which PC cells downregulate CARD9, leading to impaired DC maturation and abrogated antitumor immunity.

The immunosuppressive microenvironment of PC is shaped not only by tumor intrinsic genetic alterations but also by additional immune evasion strategies, including the secretion of immunomodulatory metabolites and dysregulated chemokines and cytokines networks^39^. Similar to our previous study, supernatants from PC cells impaired DC maturation and function, indicating that tumor derived metabolites play an important role in suppressing DC activity. Fractionation of Panc02 cell supernatants validated that small-molecule metabolites were the principal mediators to this effect. Given that enhanced glycolysis and lactate accumulation are hallmarks of various cancers, we situated lactate as a candidate metabolite and found that it promoted YY1 activation and nuclear translocation in DCs, leading to CARD9 suppression, which could be reversible by genetic or pharmacologic inhibition of LDHA.

PTMs are essential regulators of protein function and cellular responses to environmental cues. Lactate-derived histone lactylation represents a newly characterized PTM that directly links “Warburg effect” to epigenetic modification and has been implicated in regulating multiple malignancies, including bladder cancer, colorectal cancer and PC^40^-^41^. Growing interest toward non-histone lactylation has further broadened the mechanistic study of lactate in human diseases. For instance, Wang *et al*. reported that lactylation of PD-L1 at lysine 189 residue suppressed liver cancer growth by inhibiting cholesterol synthesis^42^. In line with these observations, we elucidated YY1 as a non-histone lactylation substrate and the p300 functions as its lactylation “writer”. The dynamic equilibrium of lysine lactylation (Kla) is jointly regulated by coordinated actions of “writers”, “erasers”, and “readers”^43^. Given that HDAC1-3 and SIRT1-3 serve as delactylases in cancer cells, we examined their potential involvement in YY1 modification. Molecular docking and Co-IP assays confirmed a direct interaction between YY1 and HDAC2, identifying HDAC2 as the key delactylase responsible for YY1 delactylation. Correspondingly, inhibition of HDAC2 enhanced YY1 lactylation, whereas HDAC2 overexpression reduced it. A recent study by Wang *et al*. demonstrated lysine 183 residue as a critical lactylation site in YY1, which enhanced FGF2 expression to promote retinal neovascularization^29^. Consistent with this study, we observed that YY1-K183R mutation significantly attenuated YY1 lactylation mediated by lactate. Lactylation at K183 also modulated YY1 ubiquitylation and stability, likely due to the covalent competition between lactylation and ubiquitination for the same lysine residue. Taken together, our current study reveals a novel lactate-YY1-CARD9 regulatory axis in which tumor derived lactate drives YY1 lactylation, enhances its transcriptional activity, suppresses CARD9 expression, and ultimately contributes to DC dysfunction within the pancreatic tumor microenvironment.

## Supplementary Material

Supplementary figures.

## Data Availability

The data used to support the findings of this study are available from the corresponding author upon request.
