# Supplementary material for: YY1 Lactylation Elicits CARD9 Deficiency in Dendritic Cells Promoting Pancreatic Cancer Immune Escape
Source: Int J Biol Sci. 2026 May 1;22(10):5036–53. doi: 10.7150/ijbs.131102 (PMC13215062; doi:10.7150/ijbs.131102)
Supplement: Supplementary file 1 — Supplementary figures. [file ijbsv22p5036s1.pdf]

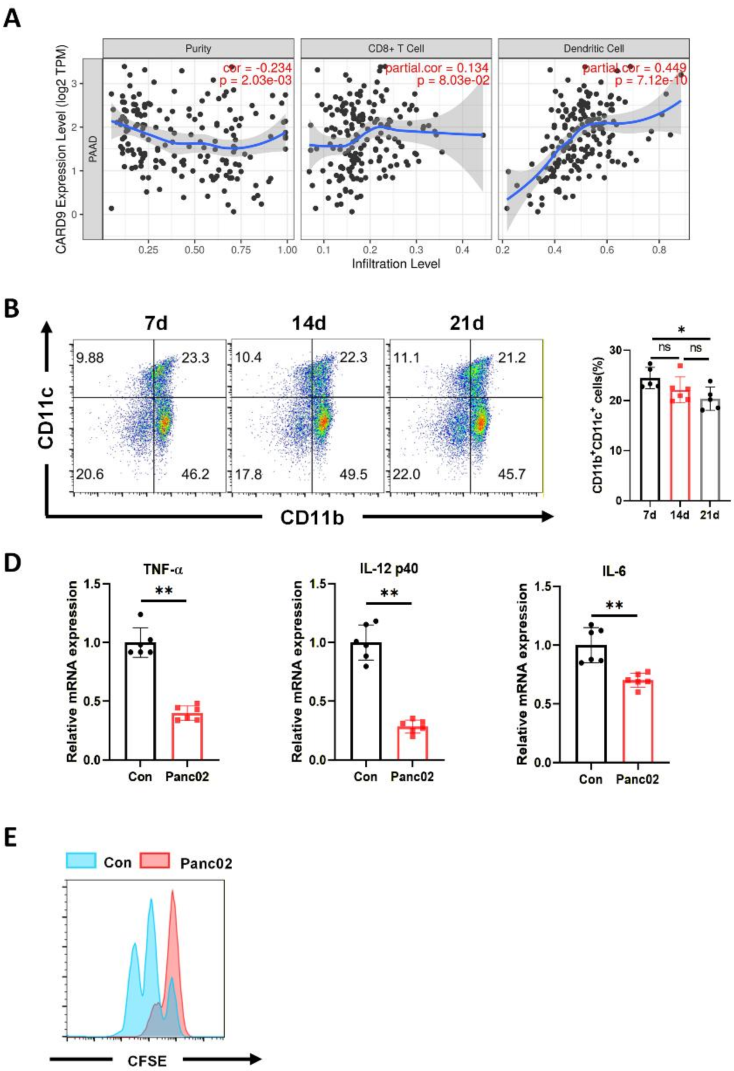

Supplementary Figure 1. A) The relationship between CARD9 and DCs and CD8$^+$ T cells was analyzed by TIDE platform. B) T-cell infiltration in tumor tissues at 7, 14 and 21 days after implantation was detected by flow cytometry. C-D) DCs co-cultured with Panc02 cells for 24 h and then given LPS and tumor antigens stimulation for 24 h. C) The levels of CARD9 mRNA were detected in DCs by qPCR. D) The levels of transcript of inflammatory factors were detected in DCs by qPCR. E) DCs that had been cultured alone or co-cultured with Panc02 cells were incubated with T cells. CFSE was used to detect T-cell proliferative capacity. All data are presented as mean ± SD, n=6. Statistical analysis was performed using the Student *t* test or by one-way ANOVA. $^{*}P < 0.05$, $^{**}P < 0.01$; ns, not significant.

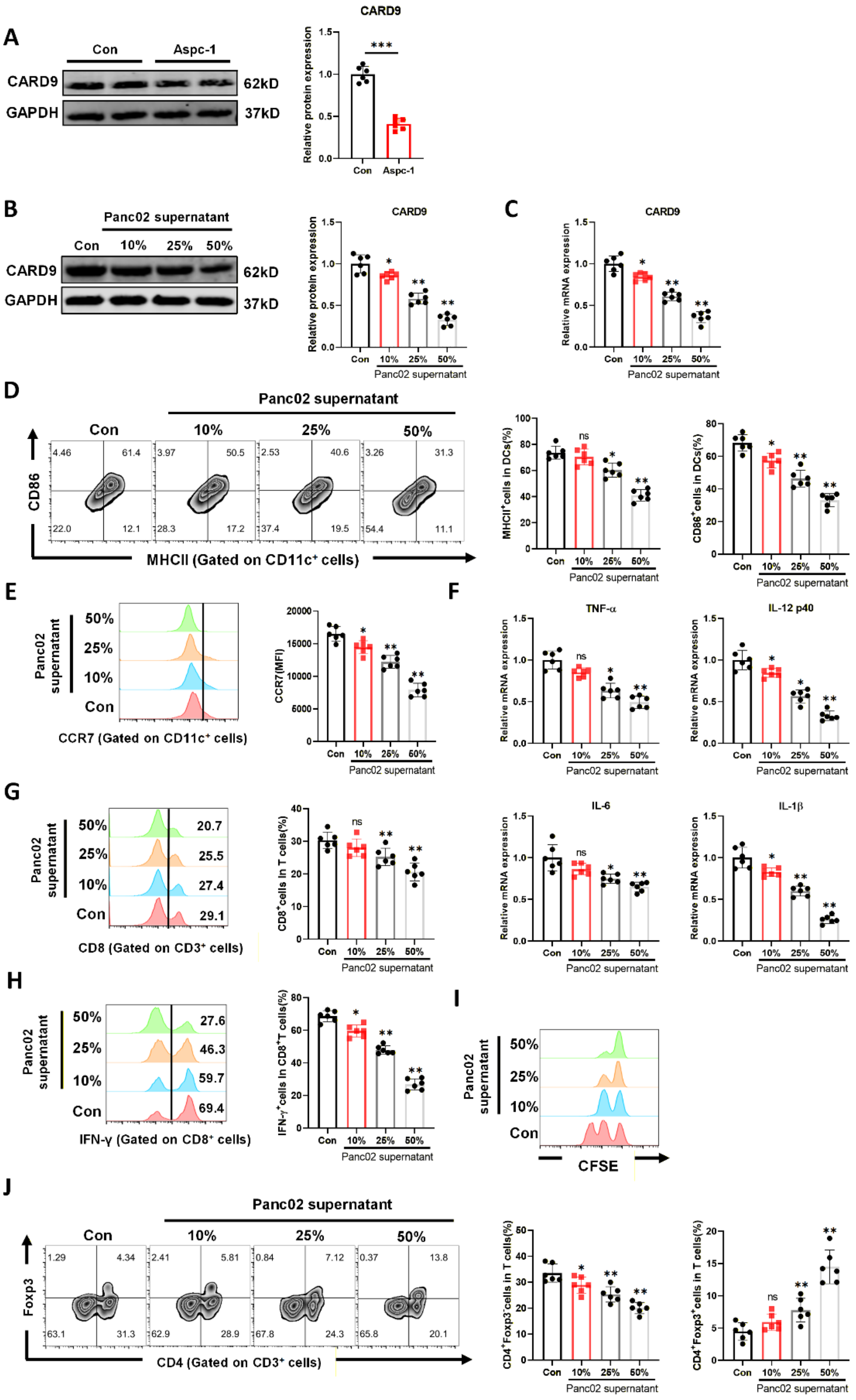

Supplementary Figure 2. A) THP-1-derived DC-like cells were co-cultured with Aspc-1 cells for 24 h and then given LPS and tumor antigens stimulation for 24 h. The levels of CARD9 protein were detected in DCs by western blot. B-F) After culturing DCs with different concentrations of

Panc02 cell-conditioned medium for 24 h, LPS and Panc02 lysates were given for 24 h stimulation. B, C) The levels of CARD9 protein and transcript were detected in DCs by western blot and qPCR techniques. D, E) CD86 and MHCII positivity as well as CCR7 levels in DCs were detected by flow cytometry. F) The levels of transcript of inflammatory factors were detected in DCs by qPCR. G-J) DCs that had been cultured alone or co-cultured with Panc02 cells were incubated with T cells. G, H) Proportion of $CD8^+$ T cells among T cells and IFN-γ positivity among them were detected by flow cytometry. I) CFSE was used to detect T-cell proliferative capacity. J) Proportion of Treg ($CD4^+Foxp3^+$) and non-Treg $CD4^+$ T cells ($CD4^+Foxp3^-$) in T cells were detected by flow cytometry. All data are presented as mean ± SD, n=6. Statistical analysis was performed using the Student *t* test or by one-way ANOVA. $^*P < 0.05$, $^{**}P < 0.01$, $^{***}P < 0.001$; ns, not significant.

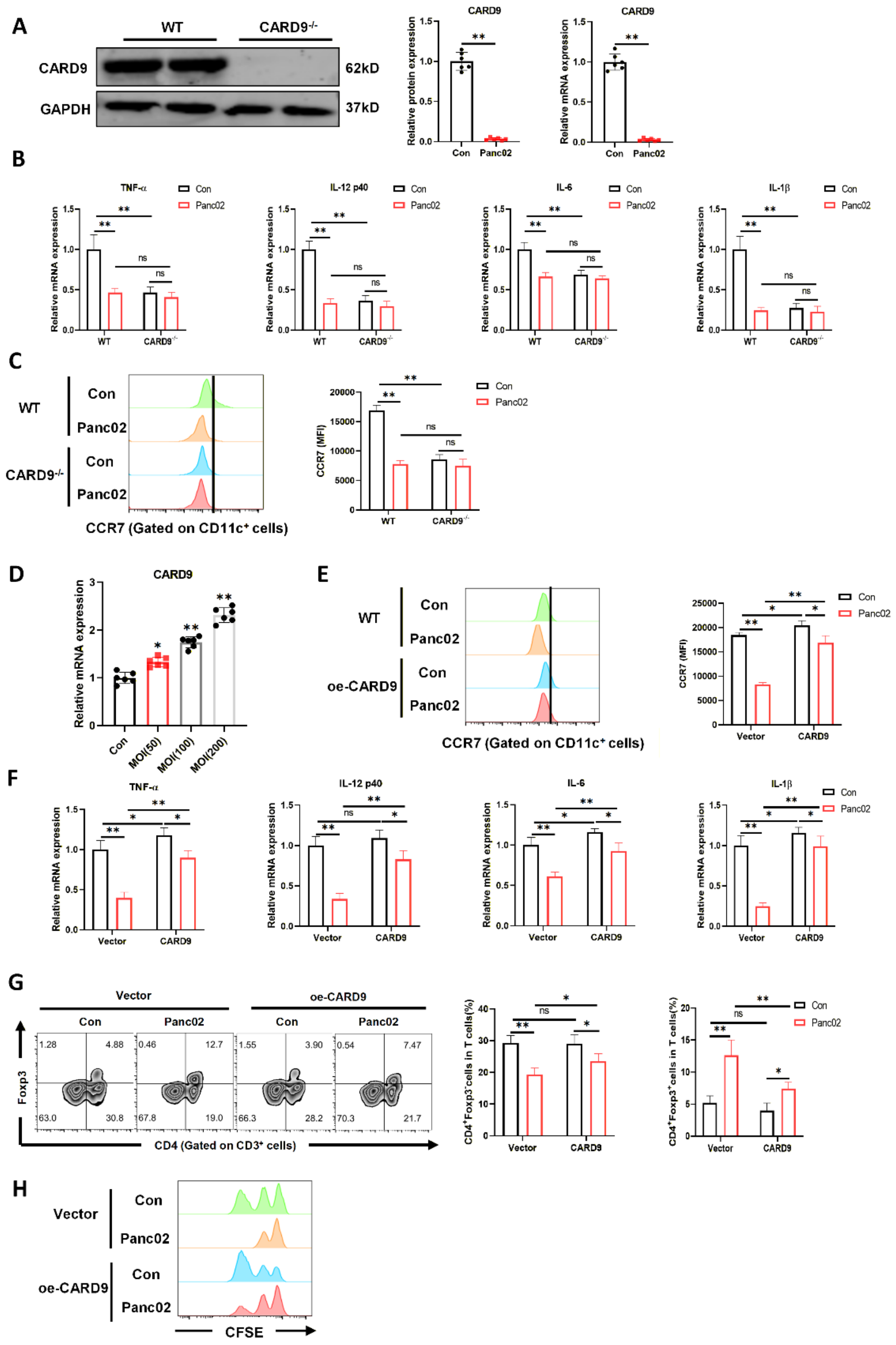

Supplementary Figure 3. A) CARD9 protein expression and transcript levels were detected by western blot and qPCR in WT-DCs and CARD9⁻/⁻ DCs. B, C) WT-DC and CARD9-/--DC were cultured alone or co-cultured with Panc02 cells and subsequently given LPS and tumor antigens stimulation. B) The levels of transcript of inflammatory factors were detected in DCs by qPCR. C) CCR7 positivity was detected in DCs by flow cytometry. D) The levels of CARD9 transcript after

DCs infected with different MOI of CARD9 overexpressing adenovirus were detected by qPCR. E, F) WT-DCs were infected with CARD9 overexpressing adenovirus (MOI=200) and vector adenovirus, and both were cultured alone or co-cultured with Panc02 cells, followed by administration of LPS and tumor antigens stimulation. E) CCR7 positivity was detected in DCs by flow cytometry. F) The levels of transcript of inflammatory factors were detected in DCs by qPCR. G, H) Then the above DCs were co-cultured with T cells for 24 h. G) Proportion of Treg ($CD4^+Foxp3^+$) and non-Treg $CD4^+$ T cells ($CD4^+Foxp3^-$) in T cells were detected by flow cytometry. H) CFSE was used to detect T-cell proliferative capacity. All data are presented as mean ± SD, n=6. Statistical analysis was performed using the Student *t* test or by two-way ANOVA. $^{*}P < 0.05$, $^{**}P < 0.01$; ns, not significant.

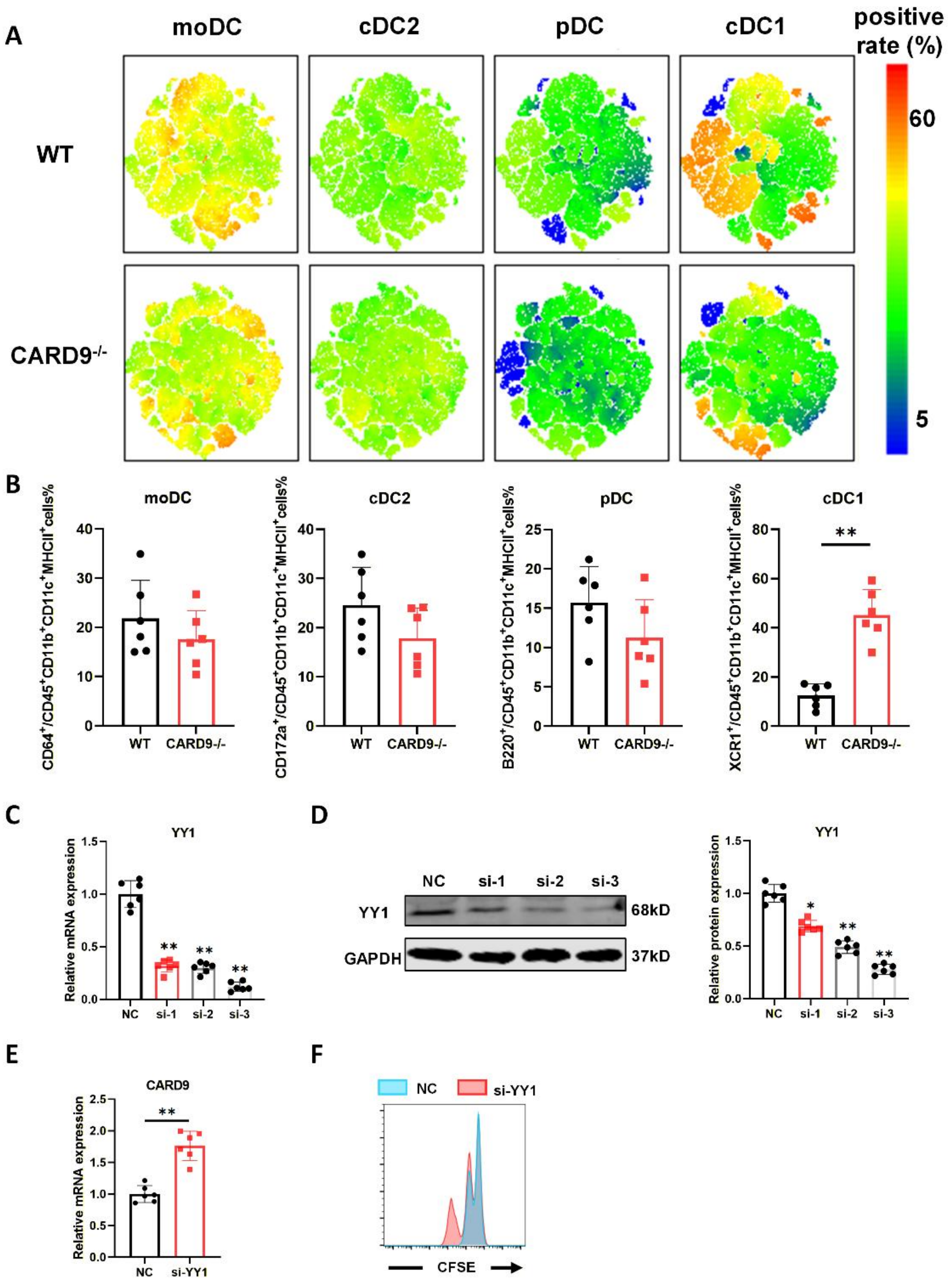

Supplementary Figure 4. A) t-SNE analysis was performed to characterize the distribution of the various DC subtypes (including cDC1, cDC2, pDC and moDC) in tumor tissues. B) The expression levels of markers corresponding to each DC subset were quantified. C) The effects of three siRNA sequences on YY1 transcript levels were detected in DCs by qPCR. D) The effects of three siRNA sequences on YY1 protein levels were detected in DCs by western blot. E) The effects of si-YY1 on CARD9 transcript levels were detected in DCs by qPCR. F) DCs transfected with NC and si-

YY1 were co-cultured with Panc02 cells and given antigenic stimulation, followed by co-culturing both DCs and T cells. CFSE was used to detect T-cell proliferative capacity. All data are presented as mean ± SD, n=6. Statistical analysis was performed using the Student t test or by one-way ANOVA. $^{*}P < 0.05$, $^{**}P < 0.01$; ns, not significant.

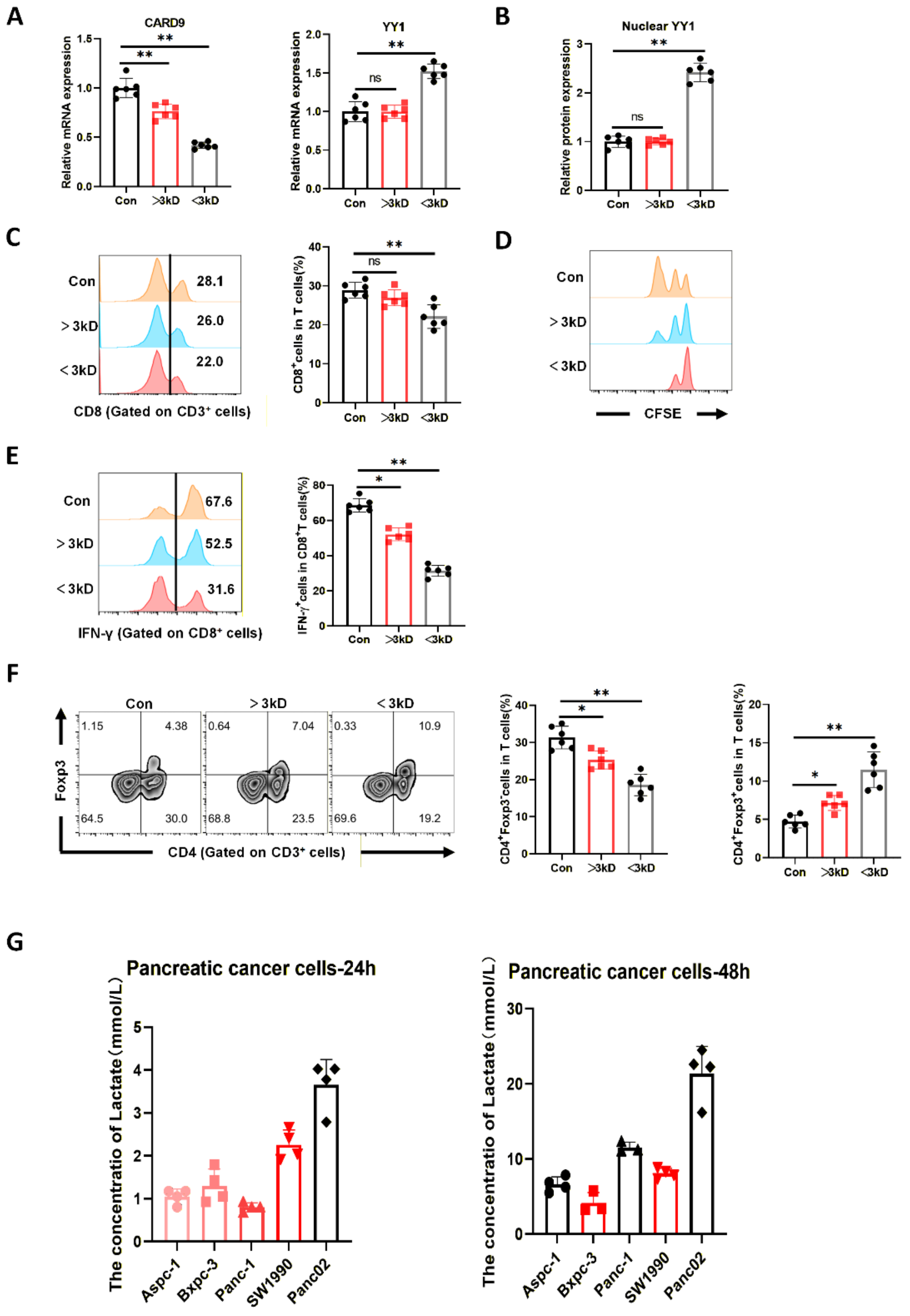

Supplementary Figure 5. A, B) Different components of the supernatant from Panc02 were separated using 3 kDa ultrafiltration centrifuge tubes, and the levels of CARD9 and YY1 total and intranuclear YY1 transcript levels were detected in DCs by qPCR after co-culturing with DCs for 24 h. C-E) After co-cultured of the above DCs with T cells, the proportion of $CD8^+$ T-cells among the T cells and their IFN-γ positivity rate among them were detected by flow cytometry. F) Proportion of Treg ($CD4^+Foxp3^+$) and non-Treg $CD4^+$ T cells ($CD4^+Foxp3^-$) in T cells were detected by flow cytometry. G) The lactate release levels of five PC cell lines at 24 h and 48 h were detected. All data are presented as mean ± SD, n=6. Statistical analysis was performed by one-way ANOVA. $^*P < 0.05$, $^{**}P < 0.01$; ns, not significant.

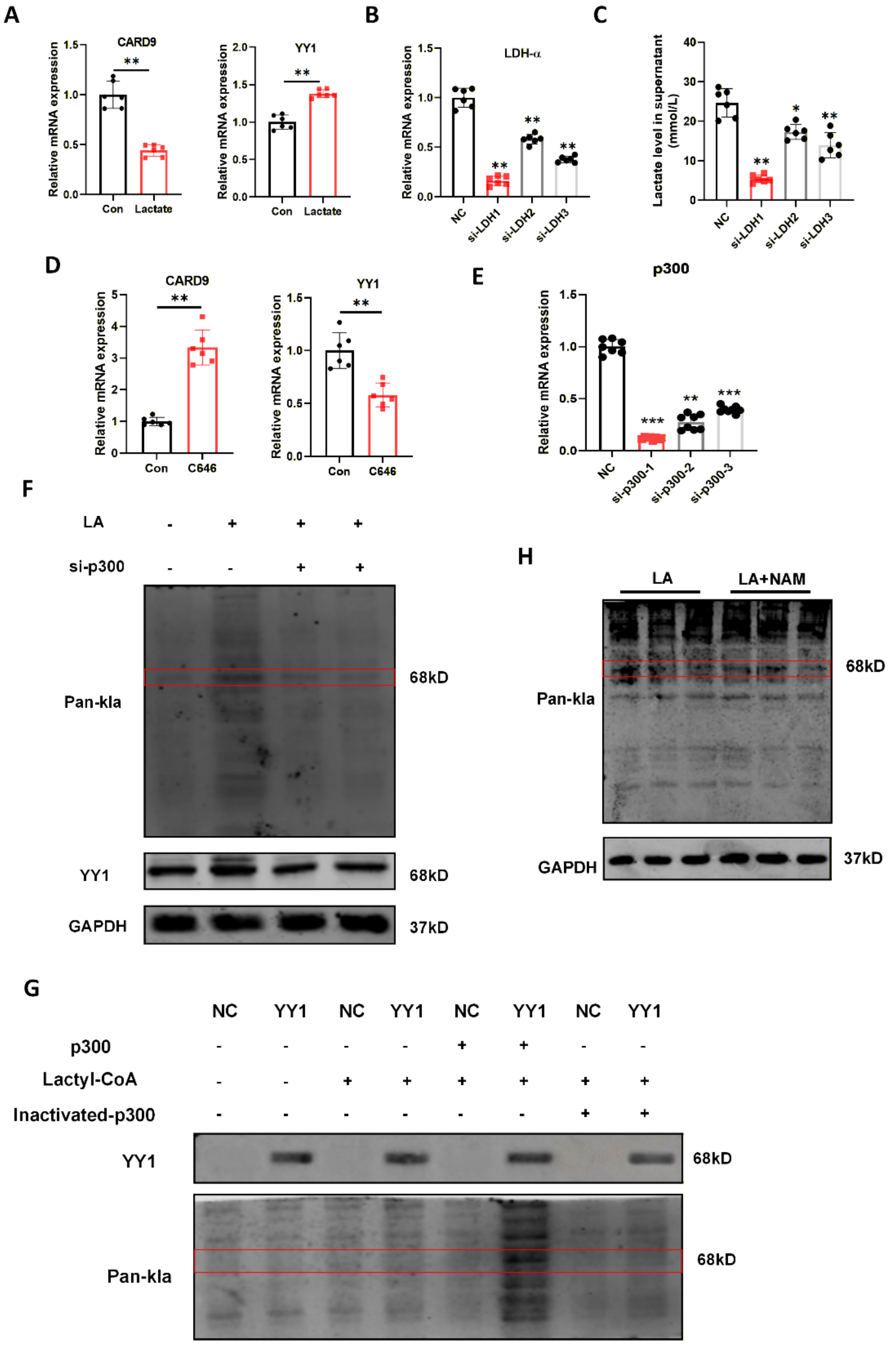

Supplementary Figure 6. A) After exogenous addition of lactate (15 mM) for 24 h, the transcript levels of CARD9 and YY1 were detected in DCs by qPCR. B) The effects of three siRNA sequences on LDH-α transcript levels were detected in DCs by qPCR. C) The lactate release levels of three siRNA sequences on LDH-α transcript levels were detected in DCs. D) CARD9 and YY1 transcript levels in DCs were detected by qPCR after treatment with the p300 inhibitor C646 (100 μM) for 24

h in the presence of lactate. E) The effects of three siRNA sequences on p300 transcript levels in DCs were detected by qPCR. F) Negative control (NC) and si-p300 were transfected into DCs separately. The levels of YY1 protein and lactylation in DCs treated with or without 15 mM lactate were detected by western blot. G) The purified YY1 was incubated with recombinant p300 (containing lactyl-CoA). The levels of YY1 protein and lactylation were detected in DCs by western blot. H) YY1 lactylation levels after treatment with SIRT inhibitor Nicotinamide (NAM, 10 mM) for 24 h in the presence of lactate were detected by western blot in DCs. All data are presented as mean ± SD, n=6/7/8. Statistical analysis was performed using the Student *t* test or by one-way ANOVA. $^{*}P < 0.05$, $^{**}P < 0.01$, $^{***}P < 0.001$.

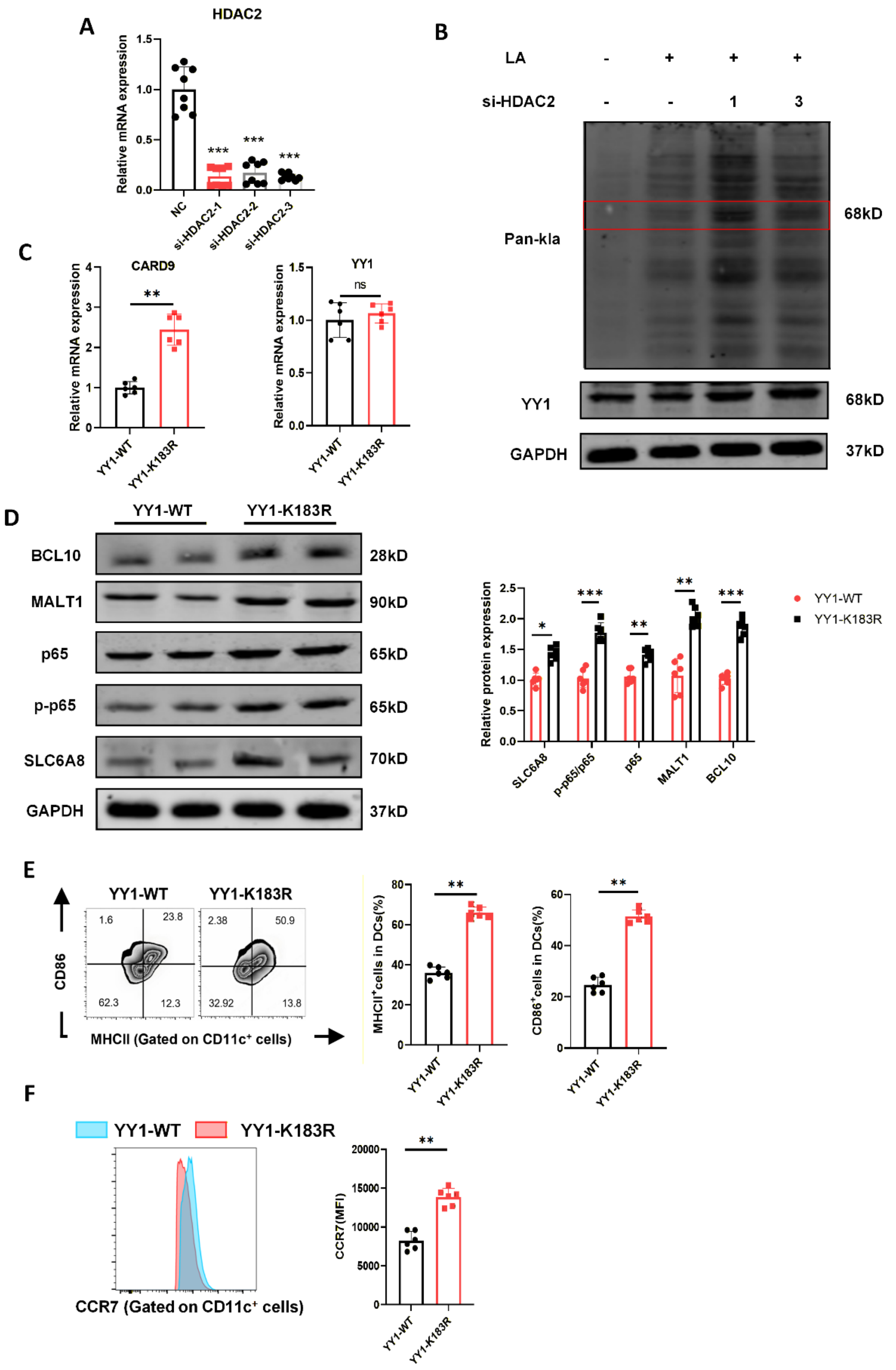

Supplementary Figure 7. A) The effects of three siRNA sequences on HDAC2 transcript levels in DCs were detected by qPCR. B) Negative control (NC) and si-HDAC2 were transfected into DCs separately. The levels of YY1 protein and lactylation in DCs treated with or without 15 mM lactate

were detected by western blot. C-F) Normal YY1 (YY1-WT) and K183R mutated YY1 (YY1-K183R, lysine residues to be mutated to arginine) plasmids were transfected into DC2.4 for 36 h, and the plasmids were treated with lactate at 12 h after transfection. C) CARD9 and YY1 transcript levels in DC2.4 after transfection were detected by qPCR. D) The levels of BCL10, MALT1, p65, p65 phosphorylation and SLC6A8 protein were detected in DC2.4 by western blot. E, F) CD86 and MHCII positivity as well as CCR7 levels in DC2.4 were detected by flow cytometry. All data are presented as mean ± SD, n=6/8. Statistical analysis was performed using the Student *t* test or by one-way ANOVA. $^{*}P < 0.05$, $^{**}P < 0.01$, $^{***}P < 0.001$; ns, not significant.
